# TRB3 links insulin/IGF to tumour promotion by interacting with p62 and impeding autophagic/proteasomal degradations

**DOI:** 10.1038/ncomms8951

**Published:** 2015-08-13

**Authors:** Fang Hua, Ke Li, Jiao-Jiao Yu, Xiao-Xi Lv, Jun Yan, Xiao-Wei Zhang, Wei Sun, Heng Lin, Shuang Shang, Feng Wang, Bing Cui, Rong Mu, Bo Huang, Jian-Dong Jiang, Zhuo-Wei Hu

**Affiliations:** 1 Immunology and Cancer Pharmacology Group, State Key Laboratory of Bioactive Substance and Function of Natural Medicines, Institute of Materia Medica, Chinese Academy of Medical Sciences, Peking Union Medical College, Beijing 100050, China; 2 Institute of Medicinal Biotechnology, Chinese Academy of Medical Sciences, Peking Union Medical College, Beijing 100005, China; 3 Institute of Basic Medical Sciences, Chinese Academy of Medical Sciences, Peking Union Medical College, Beijing 100005, China

## Abstract

High insulin/IGF is a biologic link between diabetes and cancers, but the underlying molecular mechanism remains unclear. Here we report a previously unrecognized tumour-promoting mechanism for stress protein TRB3, which mediates a reciprocal antagonism between autophagic and proteasomal degradation systems and connects insulin/IGF to malignant promotion. We find that several human cancers express higher TRB3 and phosphorylated insulin receptor substrate 1, which correlates negatively with patient's prognosis. TRB3 depletion protects against tumour-promoting actions of insulin/IGF and attenuates tumour initiation, growth and metastasis in mice. TRB3 interacts with autophagic receptor p62 and hinders p62 binding to LC3 and ubiquitinated substrates, which causes p62 deposition and suppresses autophagic/proteasomal degradation. Several tumour-promoting factors accumulate in cancer cells to support tumour metabolism, proliferation, invasion and metastasis. Interrupting TRB3/p62 interaction produces potent antitumour efficacies against tumour growth and metastasis. Our study opens possibility of targeting this interaction as a potential novel strategy against cancers with diabetes.

## Abstract (web summary)

High insulin/IGF is a biologic link between diabetes and cancer. Here, the authors show a tumour promoting mechanism for stress protein TRB3 which mediates a reciprocal antagonism between autophagic and proteasomal degradation systems and connects insulin/IGF to malignant promotion.

## Graphical abstract

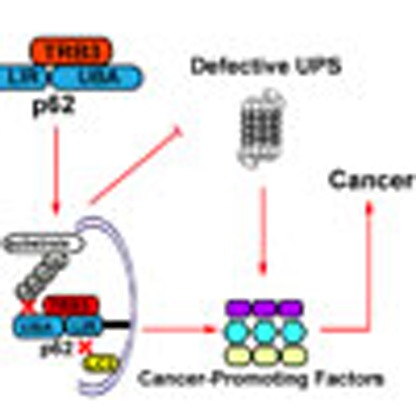

Cancer and type 2 diabetes (T2D) are two multifactorial chronic diseases with tremendous impact on health worldwide^1^,^2^. An enhanced risk for many forms of cancer has been identified in patients with obesity or T2D from clinical and epidemiologic studies^3^,^4^,^5^. Moreover, a number of studies suggest that some medications used to treat T2D result in either an increased or decreased risk of cancers depending on their either increasing or decreasing effect on hyperinsulinaemia and hyperglycaemia^6^,^7^. Indeed, cancer and T2D share a number of metabolic risk factors, among which high insulin/IGF, hyperglycaemia, glucose deprivation, hypoxia and inflammatory factors have been considered to be potential biologic links between the two diseases, but with underlined mechanisms far from understood^7^,^8^,^9^. For instance, clinical trials have shown that antagonism of the insulin/IGF signalling produces a disappointing anticancer efficacy^7^,^9^.

Recently, we found that the pseudokinase Tribbles homolog 3 (TRB3), a stress and metabolic sensor^10^,^11^,^12^, plays a crucial role in transforming growth factor (TGF)-β1-mediated cancer invasion and migration by interacting with signal molecule SMAD3 (ref. 13). This finding is consistent with a previously reported association between TRB3 expression and poor overall survival in breast cancer^14^ and colorectal cancer^15^. Given that TRB3 senses a variety of metabolic and stress signals and that the enhanced TRB3 expression leads to insulin resistance^16^,^17^,^18^, we investigate whether and how TRB3 mediates the malignancy-promoting actions of these metabolic factors. We seek to establish a TRB3-mediated mechanism connecting metabolic stresses to malignant diseases.

Here we identify TRB3 acting as a link between insulin/IGF and tumour development and progression. Metabolic stresses including Insulin/IGF-1 enhance the expression of TRB3 in a diversity of human tumour tissues and cancer cells. TRB3 depletion protects against the tumour-promoting actions of insulin/IGF in cancer cells and suppresses tumour initiation, and growth and metastasis in mice. TRB3 exerts the tumour-promoting effects through interacting with p62 and interfering with the p62 cargo function, which reciprocally induces an antagonistic effect between autophagic and proteasomal degradation. Interrupting the TRB3/p62 interaction produces potent antitumour efficacies in mice. Therefore, our study reveals a previously unrecognized tumour-promoting mechanism for stress protein TRB3 and opens the possibility of targeting this interaction as a potential strategy against cancers with diabetes.

## Results

### TRB3 mediates tumour-promoting actions of insulin/IGF

**Figure 1:**
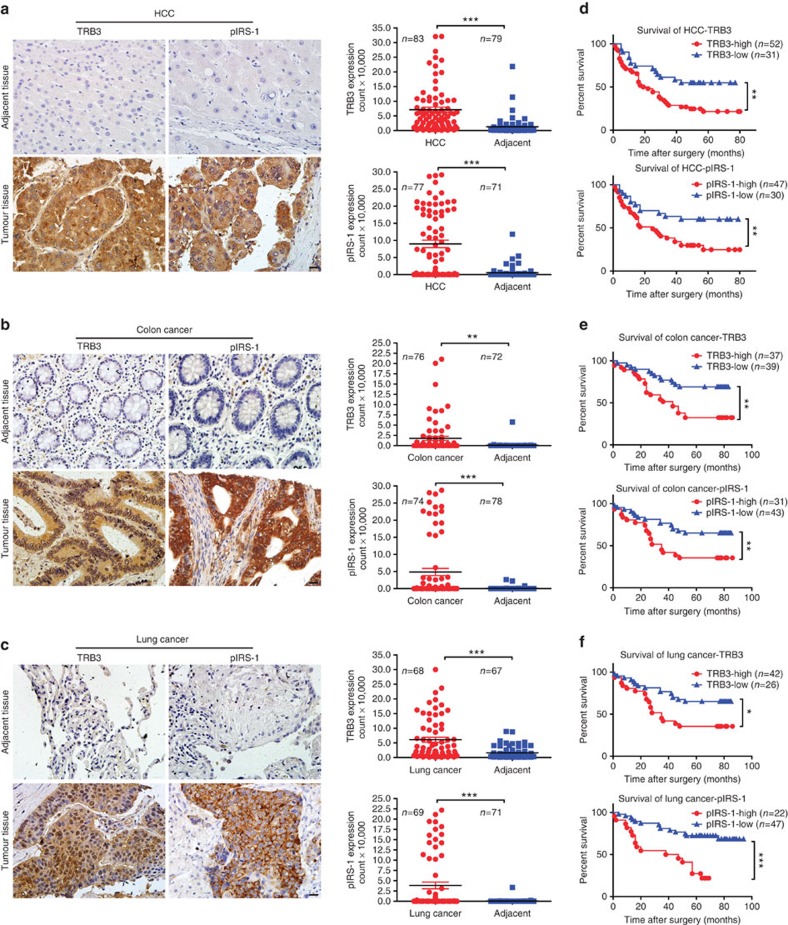
The expressions of TRB3 and pIRS-1 positively correlate with poor prognosis of human cancers. (**a**–**c**) Expression of TRB3 or pIRS-1 was detected with immunohistologic staining in tumour and adjacent non-tumour tissues of the human liver (**a**), colon (**b**) and lung (**c**). Data are representative of stained tumour and adjacent non-tumour tissues (left; scale bar, 20 μm) with quantized analyses of paired clinical samples (right). The sample size is indicated in the quantify graphs. Statistical significance was determined by Student's *t*-test; **P<0.01, ***P<0.001. (**d**–**f**) Kaplan–Meier plot of overall survival of patients with HCC (**d**), colon cancer (**e**) and lung cancer (**f**) stratified by TRB3 or pIRS-1 expression level. Tissues were scored as low (<20% TRB3 or pIRS staining for all tumour cells) and high (TRB3 or pIRS-1 staining >20% for all tumour cells or strong staining for >5% tumour cells). The sample size is indicated in the quantify graphs. A log-rank test is used for statistical analysis. *P<0.05; **P<0.01; ***P<0.001.

Using human tumour tissue microarrays with hepatocellular carcinoma (HCC), colon cancer and lung cancer, we found that higher level of TRB3 was expressed in tumour tissues than in adjacent non-tumour tissues (Fig. 1a–c). TRB3 expression correlated negatively with survival rates of patients with these cancers (Fig. 1d–f). In addition, higher expression of phosphorylated insulin receptor substrate 1 (pIRS-1) was detected in these tumour tissues than in adjacent non-tumour tissues (Fig. 1a–c) and negatively correlated with survival rates of patients with these cancers (Fig. 1d–f). Moreover, a positive correlation was observed between TRB3 and pIRS-1 levels in these tumour tissues (Supplementary Table 1). These data suggest potential roles of TRB3 and insulin/IGF signals in the progression of these cancers. Indeed, insulin and IGF-1 not only stimulated TRB3 expression in human HepG2 cells in time-dependent and concentration-dependent manners by activating TRB3 transcription (Supplementary Fig. 1a–c)^17^, but also enhanced TRB3 expression in human colon and lung cancer cells (Supplementary Fig. 1d). Interestingly, TRB3 expression was increased in response to variety of metabolic stresses, including glucose deprivation, high glucose, hypoxia or tumour-necrosis factor-α in HepG2 cells (Supplementary Fig. 1e). Insulin or IGF-1-stimulated reactive oxygen species production, γH2AX expression, increase of the S-phase fractions and apoptosis evasion; however, TRB3 depletion attenuated the effects of insulin/IGF-1 (Supplementary Fig. 1f–j). Hyperinsulinaemia exerts oncogenic actions via activation of the PI3K-AKT-mTORC and MAPK (mitogen-activated protein kinase)/ERK (extracellular signal-regulated kinase) pathways. Although silencing TRB3 did not change these signals in quiescent cells, IRS-1, PI3K-p85 and AKT were activated in response to IGF-1 (Supplementary Fig. 1k), suggesting that silencing TRB3 enhances insulin sensitivity that coincides with the observation in ref. 19. However, TRB3 depletion protected from IGF-1-induced ERK activation (Supplementary Fig. 1k) and deprived insulin/IGF-stimulated proliferation of HepG2 cells (Supplementary Fig. 1l). These data indicate that TRB3 mediates the tumour-promoting actions of insulin/IGF-1.

### Depletion of TRB3 inhibits tumour promotion

**Figure 2:**
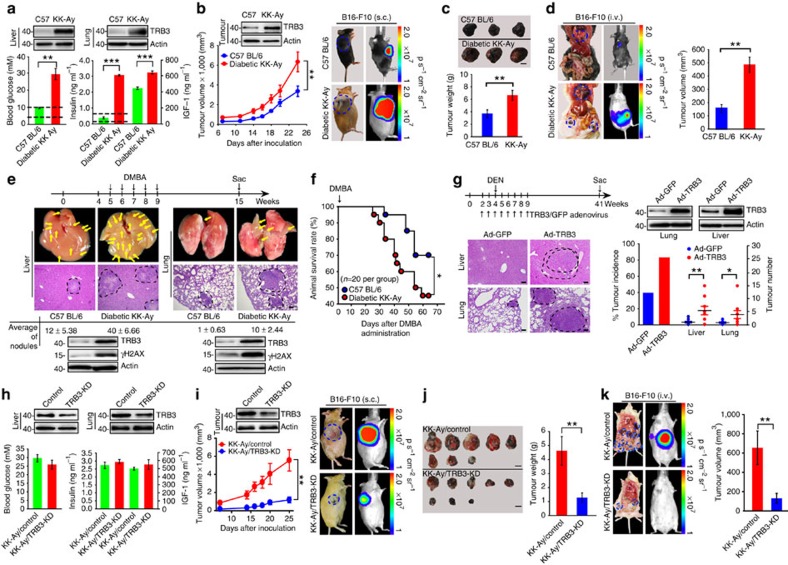
TRB3 promotes tumour development in T2D mice. (**a**) Blood glucose/insulin/IGF-1 was measured in C57 BL/6 and KK-Ay mice. Dashed lines indicate the normal range of blood glucose (3.5–9.8 mM) or insulin (0.2–0.6 ng ml^−1^). Data are mean±s.e.m. of three assays (*n*=8 per group). Inserts show TRB3 expression in the liver and lungs. (**b**) KK-Ay or C57 BL/6 mice were s.c. injected with B16-F10 cells (1.5 × 10^5^). Data are the mean volumes±s.e.m. at indicated times and representative mice (*n*=7 per group). Insert shows TRB3 expression in xenograft tumour. (**c**) Photographs of representative tumour and quantified tumour weight. Data are mean weight±s.e.m. (*n*=7 per group). Scale bar, 1 cm. (**d**) KK-Ay or C57 BL/6 mice were i.v. injected with B16-F10 cells (3 × 10^5^). Data are representative of macroscopy and bioluminescence images with total tumour volumes at multiple metastatic sites (mean±s.e.m.; *n*=12 per group). (**e**) Male KK-Ay (*n*=11) and C57 BL/6 (*n*=9) mice were p.o. gavaged with 0.1 ml (total 1 mg) DMBA once a week for 5 weeks. Data are timeline (upper) and macroscopic, and histopathological analysis of tumours (lower) with numbers of tumour nodules (mean±s.e.m.) and expression of TRB3 and γH2AX in the liver and lung. Scale bar, 10 μm. (**f**) Kaplan–Meier survival curve for mice fed with DMBA (*n*=20 per group). Statistical significance was determined by Kaplan–Meier log-rank test; *P<0.05. (**g**) Overexpression of TRB3 promotes DEN-induced tumorigenesis. Data are timeline and TRB3 expression in the liver and lungs (upper), and haematoxylin and eosin-stained sections plus incidence and number of tumour nodules (lower). Data are mean±s.e.m. (*n*=10 per group). Scale bar, 10 μm. (**h**) Blood glucose/insulin/IGF-1 was measured in mice infected with control-shRNA or TRB3-shRNA lentiviral particles (2 × 10^5^). Data are mean±s.e.m. of three assays with triplicates (*n*=8 per group). The inserts show TRB3 expression in the liver and lung. (**i**,**j**) TRB3 knockdown (*n*=9) or control KK-Ay (*n*=8) were s.c. inoculated with B16-F10 cells (1.5 × 10^5^). Data are the mean volumes±s.e.m. at indicated times and representative mice (**i**) and tumours along with tumour weight (**j**). Scale bars, 1 cm. (**k**) TRB3 knockdown (*n*=9) or control KK-Ay (*n*=8) were i.v. injected with B16-F10 cells (3 × 10^5^). Data are representatives of macroscopy and bioluminescence images with total tumour volumes (mean±s.e.m) at multiple metastatic sites. Except for **f**, statistical significance was determined with Student's *t*-test; *P<0.05; **P<0.01; ***P<0.001.

Genetically diabetic KK-Ay mouse has been widely used as a relevant model of human T2D (ref. 20). Adult KK-Ay mice not only exhibited hyperglycaemia, hyperinsulinaemia as well high plasma IGF-1, but also showed higher TRB3 expression in the liver and lungs compared with C57 BL/6 mice (Fig. 2a). We found that B16-F10 melanoma cells grew faster in diabetic KK-Ay mice than in C57 BL/6 mice, especially at the later stage after tumour-cell inoculation; the xenograft tumours from KK-Ay mice showed a higher TRB3 expression in comparison with those from C57BL/6 mice (Fig. 2b,c). Metastatic nodules with huge gross volume were found in multiple organs, including lungs, mesentery, omentum, mediastinum and axillary lymph nodes, in KK-Ay mice, whereas lungs were primary metastatic organ with smaller volume and few metastases were found in the mediastinum of C57 BL/6 mice (Fig. 2d and Supplementary Table 2). We compared tumorigenesis in C57 BL/6 and KK-Ay mice fed with 7,12-dimethylbenz(a) anthracene (DMBA), a chemical carcinogen causing Ras mutation^21^. More HCC and lung tumours with a higher mortality rate developed in KK-Ay mice than in C57 BL/6 mice (Fig. 2e,f). Consistently, higher expression of TRB3 and γH2AX was found in the liver and lungs of KK-Ay mice (Fig. 2e). We then examined whether ectopically expressed TRB3 promoted carcinogen-evoked tumorigenesis. HCC or lung tumours developed in 80% of TRB3-Ad but in less than 50% of green fluorescent protein (GFP)-Ad-infected C57 BL/6 mice in response to diethylnitrosamine (DEN; Fig. 2g). In addition, more tumour nodules developed in the TRB3-Ad than in GFP-Ad-infected mice (Fig. 2g). To validate the role of TRB3 in diabetes-accelerated tumour development, we examined tumour growth and metastasis in TRB3 knockdown KK-Ay mice. We found that TRB3-short hairpin RNA (shRNA) lentiviral infection reduced TRB3 expression but did not change hyperglycaemia and plasma level of insulin or IGF-1 (Fig. 2h). However, TRB3 knockdown repressed tumour growth and metastasis in KK-Ay mice (Fig. 2i–k). As shown in Fig. 2i, TRB3 expression in tumour nodules from TRB3 knockdown KK-Ay mice was much lower than that from control KK-Ay mice, suggesting that the higher insulin/IGF-1 in TRB3 knockdown KK-Ay mice cannot enhance TRB3 expression in the inoculated tumour cells as it does in the control KK-Ay mice. To further confirm that whether it was the reduced TRB3 in tumour cells responsible for the tumour growth inhibition, B16-F10 cells stably expressing control-shRNA or TRB3-shRNA sequences were subcutaneously (s.c.) or intravenously (i.v.) injected into KK-Ay and C57 BL/6 mice. We found that control B16-F10 cells grew at least three times faster than TRB3 depletion B16-F10 cells in KK-Ay mice (Supplementary Fig. 2a). However, control B16-F10 cells grew only two times faster than TRB3 depletion B16-F10 cells in C57 BL/6 mice (Supplementary Fig. 2c). Furthermore, control B16-F10 cells formed metastatic nodules eight times more than TRB3 depletion B16-F10 cells did in KK-Ay mice (Supplementary Fig. 2d); however, control B16-F10 cells form metastatic nodules only three times more than TRB3 depletion B16-F10 cells inC57 BL/6 mice (Supplementary Fig. 2b). These data suggest that it is the insulin/IGF-induced TRB3 in tumour cells that is responsible for the tumour promotion actions. We further evaluated the metastatic and proliferative effects of cancer cells with TRB3 depletion in BALB/c nude mice. Mice i.v. inoculated with HepG2 cells expressing control-shRNA developed more metastatic nodules than mice with cells expressing TRB3-shRNA1/2 (Supplementary Fig. 3a,b). Thus, mice with TRB3-silenced cells survived much longer than did mice with TRB3-control cells (Supplementary Fig. 3c). Moreover, mice s.c. inoculated with control-shRNA cells developed larger tumours than mice with TRB3-shRNA1/2 cells (Supplementary Fig. 3d–f). Similarly, TRB3 depletion inhibited metastasis and growth of HCT-8 cells (Supplementary Fig. 3g,h). These results suggest that TRB3 plays a crucial role in tumorigenesis, tumour growth and metastasis in mice, particularly in mice with T2D.

### TRB3 causes p62 accumulation and attenuates autophagic flux

**Figure 3:**
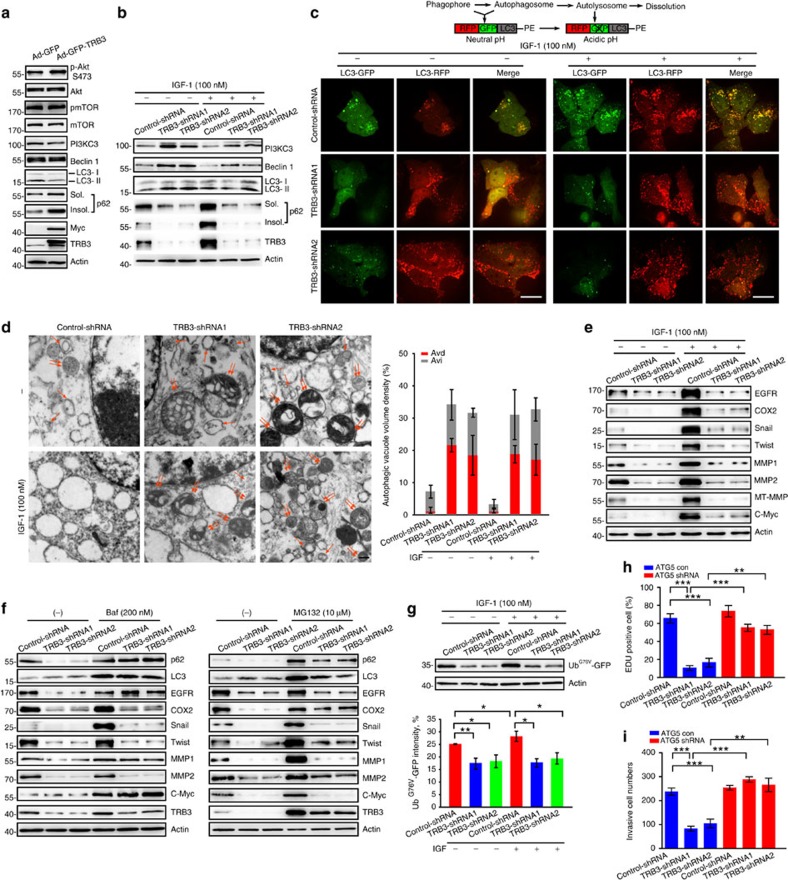
Enhanced TRB3 compromises selective autophagy and clearance of UPS. (**a**) BEAS-2B cells were infected with TRB3-Myc-adenovirus expressing Myc-Tagged TRB3 with an independently expressing GFP. GFP-adenovirus was used as control. Indicated molecules were detected by immunoblotting. Data are representatives of four independent assays. (**b**) TRB3-silenced cells were treated with IGF-1 (100 nM) for 12 h and indicated proteins were detected by immunoblotting. Data are representatives of four independent assays. (**c**) Control or TRB3-silenced HepG2 cells were infected with adenovirus with mRFP-GFP-LC3-PE. After 24 h, the cells were treated with IGF-1 (100 nM) for 12 h and the flux rate of autophagy was detected with Live Cell Imaging Microscopy. Data are schematic drawing of the autophagy process (upper) and microscopy images showing red-coloured autophagolysosomes or red/green double-coloured autophagosomes (lower). Scale bar, 10 μm. See also Supplementary Movies 1–6. (**d**) Silencing TRB3 increased the number of autophagosomes (Avi) and autolysosomes (Avd) in HepG2 cells treated with or without IGF-1 (100 nM). Data are representative images of TEMs of three independent assays. Single arrows denote Avi; double arrows indicate Avd. Scale bar, 500 nm. The ratio of autophagic vacuole area to the cytoplasmic area was determined by morphometric analysis. Data are means±s.e.m. (**e**) TRB3-silenced cells were treated with IGF-1 (100 nM) for 12 h. Expression of indicated proteins was detected by immunoblotting. Data are representatives of four independent assays. (**f**) TRB3-silenced HepG2 cells were treated with bafilomycin (left) or MG132 (right) for 12 h. Indicated proteins were detected by immunoblotting. Data are representatives of four independent assays. (**g**) TRB3-silenced or control cells were transfected with Ub^G76V^-GFP plasmid. The cells were treated with or without IGF-1 for 12 h and the expression of Ub^G76V^-GFP was immunoblotted with anti-GFP antibody (Ab; left) or detected with flow cytometry (right). Data are means±s.e.m. (*n*=4). (**h**,**i**) HepG2 cells stably expressing TRB3-shRNA and ATG5-shRNA simultaneously were generated. The proliferative and invasive capacities of cells were evaluated with Edu (**h**) and transwell assays (**i**). Statistical significance was determined with one-way analysis of variance (ANOVA); *P<0.05; **P<0.01; ***P<0.001.

Because TRB3 knockdown enhances phosphorylation of AKT, a negative modulator of autophagy, in response to IGF^19^,^22^, we examined the effect of changed TRB3 level on autophagic signals in cancer cells. Overexpression of TRB3 did not activate AKT and mTOR but inhibited LC3-I/II conversion and increased expression of both soluble and insoluble p62, a selective autophagy receptor, in BEAS-2B cells (Fig. 3a). In contrast, silencing TRB3 not only activated Beclin1, PI3KC3 and LC3-I/II but also decreased expression of soluble and insoluble p62 in HepG2 cells (Fig. 3b). Moreover, TRB3 depletion protected against IGF-1-suppressed autophagy and p62 accumulation (Fig. 3b and Supplementary Fig. 4a), suggesting that TRB3 mediates the IGF-1-induced p62 accumulation and autophagy inhibition. Similarly, insulin and IGF-1, no matter they activate or not the autophagic signals, induced a significant accumulation of insoluble p62 in human liver, colon and lung cancer cells (Supplementary Fig. 4b). KK-Ay mice with high TRB3 expression showed accumulation of soluble and insoluble p62, whereas TRB3-depleted KK-Ay mice showed reduced p62 levels in liver, lung and xenograft tumours (Supplementary Fig. 4c). Because the p62 level is one of critical indicators of autophagic flux^23^,^24^, these findings suggest that enhanced TRB3 causes p62 accumulation and attenuates autophagic flux.

To verify the inhibitory role of TRB3 in control of the autophagic flux, the flux rate of autophagy was measured with live-cell imaging using an mRFP-GFP-LC3 reporter construct. We found that more ectopically expressed mRFP-GFP-LC3 was detected as red and yellow speckles in IGF-1-treated control-shRNA cells than in untreated-control-shRNA cells. However, dynamically moving red spots with occasional yellow spots were observed in TRB3-shRNA cells and IGF-1-treated TRB3-shRNA cells (Fig. 3c and Supplementary Movies 1–6), indicating that TRB3 mediates the IGF-1-induced accumulation of autophagosomes. Transmission electron microscopy (TEM) revealed that silencing TRB3 increased number of autophagosomes (Avi) and autolysosomes (Avd) in HepG2 cells treated with or without IGF (Fig. 3d). These data suggest that TRB3 mediates the IGF-suppressed autophagic flux, whereas silencing TRB3 induces the autophagic flux.

The effect of TRB3 on the cell-wide protein stability was evaluated in TRB3-silenced HepG2 cells with an antibody array containing 1,000 proteins. We found that 66.7% of the 1,000 proteins had no change in the expression level or with low signal intensity; 6.8% of the proteins were downregulated; and 26.5% of the proteins were upregulated in TRB3-silenced cells (Supplementary Fig. 4d, upper), suggesting that silencing TRB3 does not change bulk degradation. Interestingly, most of the downregulated proteins were tumour-promoting factors in TRB3-silenced cells (Supplementary Table 3). For instance, expression of EGFR, a critical protein implicated in tumour growth, metabolism and dissemination^25^,^26^, was reduced by over fivefold in TRB3-silenced cells (Supplementary Fig. 4d, lower). Silencing TRB3 not only decreased the basal level of critical tumour-promoting factors such as EGFR, COX2, MMP1, MMP-2, MT-MMP, Snail, Twist and c-Myc but also protected them from the IGF-1 induction (Fig. 3e). These data indicate that TRB3 acts as a proto-oncogene rendering the cancer cells a feature of cancer-initiating phenotype^27^.

To determine the mechanism for the accelerated degradation of tumour-promoting factors and verify the induction of autophagic flux in TRB3-depleted cells, a turnover assay was conducted in the presence or absence of bafilomycin, a later-phase autophagy inhibitor. Turnover of LC3, EGFR, c-Myc, MMP1 and p62 was inhibited in the presence of bafilomycin (Fig. 3f). However, turnover of COX2, Twist and MMP-2 was not inhibited by bafilomycin rather than by proteasome inhibitor MG132 (Fig. 3f), suggesting that TRB3 interferes with the substrate clearance by compromising both autophagy and ubiquitin–proteasome system (UPS). Notably, turnover of p62, LC3 and TRB3 was repressed by both autophagy and UPS inhibitors. Turnover of Snail was inhibited by neither bafilomycin nor MG132 (Fig. 3f) because Snail is regulated at the transcription level in TRB3-silenced cells^13^. The expression of these tumour-promoting factors was also higher in KK-Ay mice than that in C57 BL/6 mice (Supplementary Fig. 4e,f). Moreover, TRB3 depletion not only reduced basal expression but also protected from IGF-1-enhanced expression of Ub^G76V^-GFP, a reporter for determining UPS activity (Fig. 3g)^28^. These data suggest that TRB3 causes accumulation of the tumour-promoting factors by impeding both autophagy and UPS in cancer cells. To validate the dysfunctions of autophagic and UPS clearance as molecular mechanisms connecting TRB3 to insulin/IGF-induced tumour development, we examined whether the inhibition of autophagy or UPS might reverse the antitumour effects of TRB3 depletion. We found that knocking down of ATG5, an essential autophagy gene, reverses the antitumour effect of TRB3 depletion in tumour cells as demonstrated by the 5-Ethynyl-2′-deoxyuridine (Edu) proliferation assay (Fig. 3h) and the transwell invasion assay (Fig. 3i). Pharmacological inhibition of autophagy or proteasome by 3-MA or MG132 could separately overturn the antitumour actions of TRB3 deficiency too (Supplementary Fig. 4g,h).

**Figure 4:**
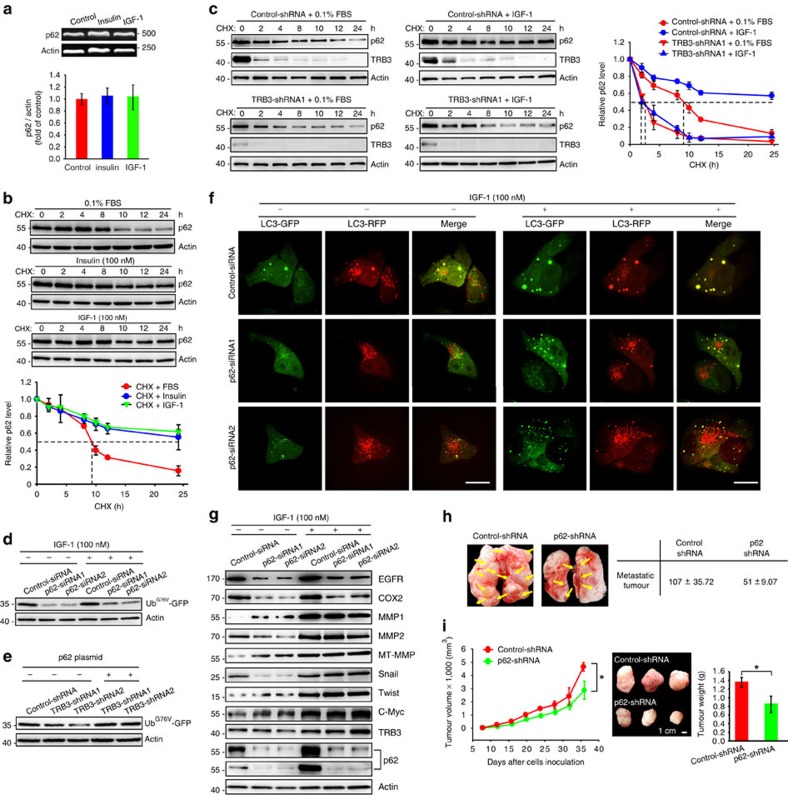
TRB3 mediates insulin/IGF-induced p62 accumulation. (**a**) HepG2 cells were treated with indicated stimulators for 12 h. The mRNAs were determined with RT–PCR using specific primer sets. Data are means±s.e.m of three independent assays. (**b**) HepG2 cells were incubated with cycloheximide (CHX) (10 μg ml^−1^) for indicated time points after insulin/IGF-1 stimulation. Indicated proteins were detected with immunoblotting. Data are means±s.e.m of three independent assays. (**c**) Control or TRB3-silenced cells were incubated with CHX (10 μg ml^−1^) for indicated time points after IGF-1 stimulation. Cell lysates were isolated for immunoblotting. Data are means±s.e.m of three independent assays. (**d**) HepG2 cells expressing p62- or control-siRNA were co-transfected with Ub^G76V^-GFP. The cells were treated with IGF-1 for 12 h. The cell lysates were isolated for immunoblotting. Data are representative of immunoblots of three independent assays. (**e**) Cells expressing control-shRNA or TRB3-shRNAs were transfected with Ub^G76V^-GFP plasmid plus or minus with p62-DDK-expressing plasmid. The lysates were isolated for immunoblotting. Data are representative immunoblots of three independent assays. (**f**) HepG2 cells expressing p62- or control-siRNA were infected with adenovirus containing mRFP-GFP-LC3-PE. After 24 h, the cells were treated with IGF-1 for 12 h, and the flux rate of autophagy was detected with Live Cell Imaging Microscopy. Data are representative images of three independent assays. Red colour shows autophagolysosomes and double-colour red/green shows autophagosomes. Scale bar, 10 μm. (**g**) Control or p62-silencing cells were treated with or without IGF-1 for 12 h. Indicated proteins were evaluated with immunoblotting. Data are representative immunoblots of three independent assays. (**h**) BALB/c nude mice were i.v. injected with HepG2 cells expressing control-shRNA or p62-shRNA (3 × 10^6^). Data are representative of number of metastases in lungs and lungs. Metastatic nodules were indicated by arrows (*n*=8 per group). (**i**) BALB/c nude mice were s.c. inoculated with HepG2 cells expressing control- or p62-shRNA (1.5 × 10^6^). Data are the mean volumes±s.e.m. at indicated times and representative tumours along with tumour weight (*n*=8 per group). Scale bar, 1.5 cm. Statistical significance was determined with Student's *t*-test; *P<0.05.

We examined how TRB3 mediated the insulin/IGF-induced p62 accumulation. Insulin/IGF-1 did not change p62 transcription (Fig. 4a) but rather markedly enhanced the half-life of p62 degradation from 8.5 to 24 h (Fig. 4b). Silencing TRB3 promoted p62 degradation in the presence or absence of IGF-1 (Fig. 4c), indicating that TRB3 mediates insulin/IGF-induced p62 accumulation. Interestingly, p62 depletion not only reduced basal but also blocked IGF-1-induced expression of Ub^G76V^-GFP (Fig. 4d), coinciding with a report that p62 accumulation by autophagy inhibition slows down the UPS-specific substrate clearance^29^. To verify whether inhibitory effect of TRB3 on UPS was mediated by p62 accumulation, the p62 level was recovered by ectopic expression of p62 in TRB3-silenced cells. Overexpressing p62 rescued Ub^G76V^-GFP activity in TRB3-silenced cells, indicating that TRB3 blocks UPS clearance through inducing p62 accumulation (Fig. 4e). In addition, silencing p62 moderately enhanced autophagic flux in quiescent cells and partially restored the IGF-1-suppressed autophagic flux (Fig. 4f). However, silencing p62 had a mixed effect on expression of the tumour-promoting factors in quiescent cells, which was quite different from the effects induced by TRB3 depletion; it could not protect them from the IGF-1 induction as did by TRB3 depletion (Fig. 4g). Thus, p62 depletion produced only a moderate antitumour role in tumour metastasis and growth (Fig. 4h,i). These results indicate that TRB3 mediates the IGF-induced p62 accumulation, which compromises UPS as well as (partially) autophagic degradation in cancer cells.

### TRB3 interacts with p62 to hinder the cargo functions of p62

**Figure 5:**
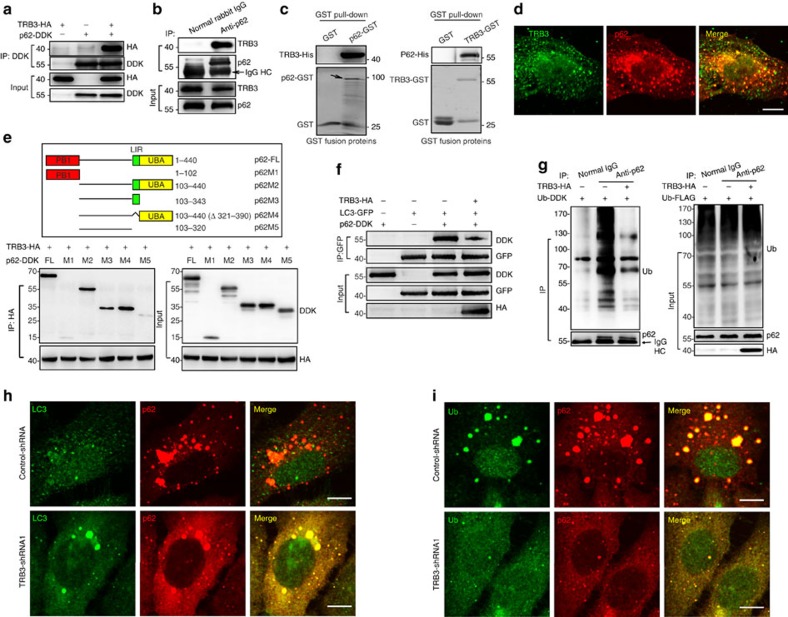
TRB3 interacts with p62 disturbing the p62 cargo functions. (**a**) HEK 293T cells were co-transfected with the expression plasmids of p62-DDK and TRB3-HA. Cell extracts were IP with anti-DDK Ab and blotted with anti-HA Ab. Data are representative immunoblots of five independent assays. (**b**) HepG2 extracts were IP with anti-p62 Ab or rabbit IgG and were blotted with anti-TRB3 Ab. Data are representative immunoblots of five independent assays. (**c**) *In vitro* interaction of TRB3/p62 was detected with GST pull-down assay. Retrieved proteins were examined by immunoblotting. GST-only protein was used as negative control. GST fusion proteins were stained with Coomassie Blue. Data are representative immunoblots of three independent assays. (**d**) Colocalization of TRB3 and p62 was detected by immunostaining. Data are representative images of three independent assays. Scale bar, 10 μm. (**e**) Mapping of p62 regions involved in TRB3 binding. Top: deletion mutants of p62. Below: HEK 293T cells were co-transfected with indicated constructs of p62 and TRB3-HA. Cell extracts were IP with anti-HA Ab. Data are representative immunoblots of three independent assays. (**f**) HEK 293T cells were transiently transfected with indicated plasmids. The p62-LC3 binding was evaluated with IP assay. Data are representative immunoblots of three independent assays. (**g**) HEK 293T cells were transfected with Ub-DDK plus or minus TRB3-HA plasmids. The binding of p62-ubiquitinated protein was evaluated with IP assay. Data are representative immunoblots of three independent assays. (**h**,**i**) The colocalization of P62/LC3 (**h**) or p62/ubiquitinated proteins (**i**) was detected by immunostaining. Scale bar, 7.5 μm. Data are representatives of three assays.

Cargo receptor p62 is a multidomain protein and can interact with LC3 for autophagic degradation of ubiquitinated and non-ubiquitinated substrates^30^,^31^. We assumed that TRB3 induced p62 accumulation and dysfunction by interacting with p62. We found that endogenous or overexpressed p62 could be co-immunoprecipitated (IP) with TRB3 (Fig. 5a,b), which was confirmed using glutathione *S*-transferase (GST) pull-down assays (Fig. 5c) and the TRB3/p62 colocalization in HepG2 cells (Fig. 5d). The binding of p62 with prokaryotic expressed TRB3-GST fusion protein (Fig. 5c) indicated that the p62–TRB3 interaction is independent of TRB3 ubiquitination. To map the TRB3 interaction region, the deletion mutants of DDK-tagged p62 were made and subjected to IP with TRB3-HA. We found that TRB3 interacted with the LIR motif and UBA domain of p62 (Fig. 5e), suggesting TRB3/p62 interaction interfering with the p62-mediated autophagy. Indeed, ectopic expression of TRB3 reduced the association of P62 with both LC3 and ubiquitinated proteins (Fig. 5f,g), whereas TRB3 depletion increased p62/LC3 or p62/Ub colocalization with a reduction in p62 and ubiquitin-positive proteins (Fig. 5h,i). These data indicate that TRB3 interacts with p62 and interferes with the binding of p62 to LC3 and to ubiquitinated proteins, leading to the dysfunction of p62-mediated autophagy and the reduced degradation of ubiquitinated proteins and p62 itself. Indeed, expression of TRB3 and p62 was increased in human HCC, colon and lung cancer tissues in comparison with normal liver, colon and lung tissues (Supplementary Fig. 5a and Supplementary Table 4). These tumour tissues showed an enhanced interaction of TRB3/p62 (Supplementary Fig. 5b) as well TRB3/p62 colocalization (Supplementary Fig. 5c), demonstrating that the enhanced TRB3/p62 interaction occurs in human cancers.

**Figure 6:**
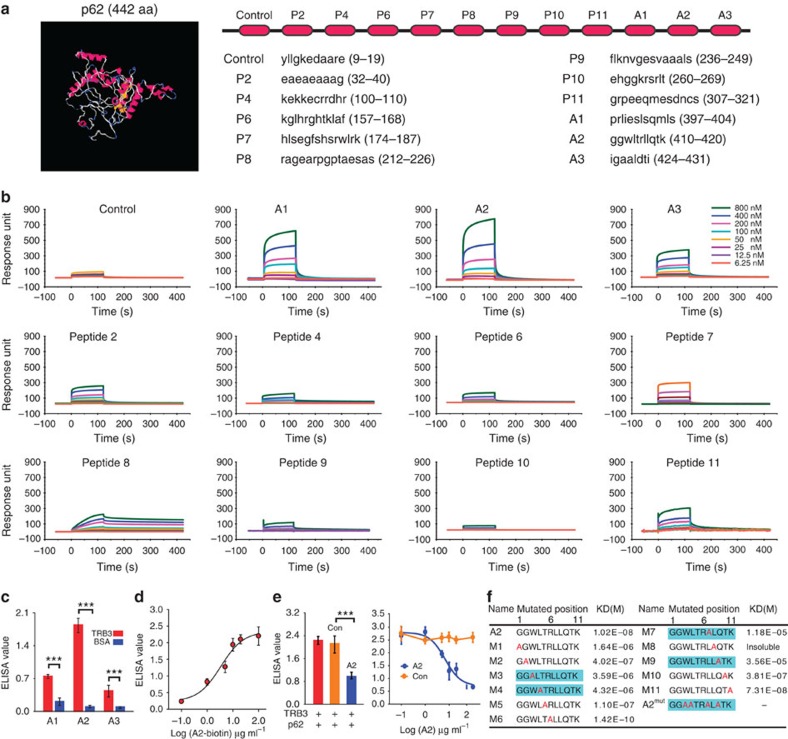
The TRB3-binding α-helical peptides are identified with the SPR screening. (**a**) The 3D structure of P62 was predicted by the I-TASSER server and the amino-acid sequences of the indicated α-helical peptides from P62 were shown. (**b**) The indicated concentrations of the peptides were passed over immobilized TRB3 on CM5 sensor chips. (**c**) ELISA analysis of the binding ability of HRP-conjugated A1, A2 and A3 (10 μg ml^−1^) to purified TRB3 protein and a negative control BSA. Data are means±s.e.m. of three independent assays. Statistical significance was determined with Student's *t*-test; ***P<0.001. (**d**) The indicated concentration of HRP-conjugated A2 was incubated with TRB3, and the binding of A2/TRB3 was measured at 450 nm after reaction with HRP substrates. Data are means±s.e.m. of three independent assays. (**e**) The p62 binding to TRB3 was competed with 0.1–100 μg ml^−1^ of A2 or control peptide, and the binding of TRB3/p62 was detected using ELISA assay. Data are means±s.e.m. of three independent assays. (**f**) Each amino acid of A2 was substituted with alanine. Kinetic interactions of mutated peptides and TRB3 were determined by SPR analyses.

Given the stress-induced TRB3/p62 interaction promotes tumour growth and metastasis, interrupting this interaction might produce an antitumour efficacy. Because most α-helical epitopes occur in protein–protein interactions (PPIs) and several short α-helical segments act as inhibitors of PPIs^32^,^33^, a series of α-helical peptides disturbing the TRB3/p62 interaction were screened with the I-TASSER server for prediction of p62 secondary structure (Fig. 6a). We found that peptide segments from the p62 UBA domain displayed a better binding to TRB3 than other α-helical peptides (Fig. 6b). However, only horseradish peroxidase (HRP)-conjugated A2, but not HRP-conjugated A1 or A3, bound to TRB3 with high affinity (Fig. 6c,d). In addition, A2 inhibited the binding of p62 to TRB3 in a concentration-dependent manner (Fig. 6e). To assess the contribution of A2 amino-acid sequence to A2/TRB3 binding, each amino-acid residue of A2 was substituted with alanine. The residues 3, 4, 7 and 9 of A2 were critical for the binding of A2 to TRB3 because the mutations abolished A2/TRB3 binding (Fig. 6f).

### Disturbing TRB3/p62 interaction inhibits tumour promotion

**Figure 7:**
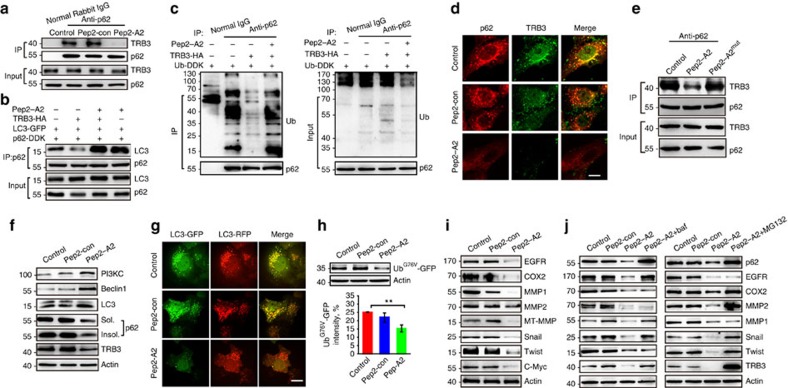
An α-helical peptide of p62 interrupts the TRB3/p62 interaction and promotes autophagy and UPS degradation. (**a**) HepG2 cells were treated with Pep2–A2 or Pep2-con (5 μM) for 12 h, and extracts were IP with anti-p62 Ab or normal rabbit IgG and blotted with anti-TRB3 Ab. (**b**,**c**) HEK 293T cells were transfected with the indicated plasmids. The effect of Pep2–A2 on the LC3/p62 and Ub/p62 binding was evaluated with Co-IP assay. (**d**) HepG2 cells were treated with Pep2–A2 or Pep2-con for 24 h, and the colocalization of the P62/TRB3 was examined by immunostaining. Scale bar, 10 μm. (**e**) HepG2 cells were treated with Pep2-con, Pep2–A2 or Pep2–A2^mut^ (5 μM) for 12 h and cell extracts were IP with anti-p62 Ab and blotted with anti-TRB3 Ab. (**f**) HepG2 cells were treated with Pep2–A2 or Pep2-con for 24 h and the autophagy-associated proteins were detected by immunoblotting. (**g**) HepG2 cells infected with mRFP-GFP-LC3 plasmid were treated with Pep2–A2 or Pep2-con for 24 h, and the images were captured with confocal microscopy. Scale bar, 10 μm. See also Supplementary Movies 4–6 for details. (**h**) HepG2 cells transfected with Ub^G76V^-GFP plasmid were treated with Pep2–A2 or Pep2-con for 24 h. Cell lysates were collected for immunoblotting analysis. (**i**) HepG2 cells were treated with Pep2–A2 or Pep2-con for 24 h. The expressions of indicated proteins were detected by immunoblotting. (**j**) HepG2 cells were treated with Pep2-con, Pep2–A2, Pep2–A2 plus bafilomycin (left) or Pep2–A2 plus MG132 (right) for 12 h. The expression of indicated proteins was detected by immunoblotting. For all panels, *n*=3 independent experiments. Data indicate mean±s.e.m. Statistical significance was determined with one-way ANOVA; **P<0.01.

**Figure 8:**
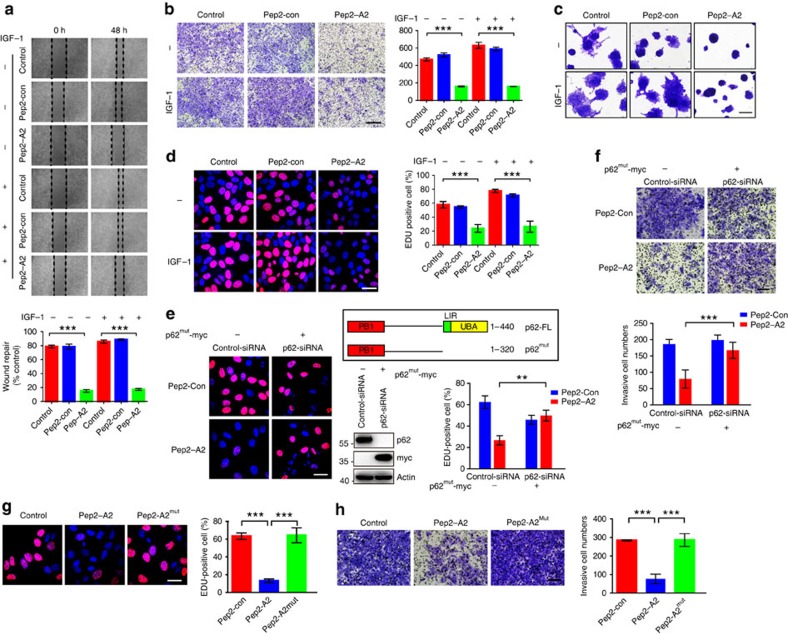
Interrupting the TRB3/p62 interaction inhibits tumour proliferation and invasion. (**a**) Wound-healing assay of HepG2 cells treated with Pep2–A2 or Pep2-con plus or minus IGF-1 at indicated times. (**b**,**c**) Pep2–A2 attenuates IGF-1-induced invasion of HepG2 cells. Data are representative transwell; scale bar, 100 μm (**b**). 3D Matrigel culture assay, scale bar, 100 μm (**c**). (**d**) Pep2–A2 attenuates IGF-1-induced proliferation of HepG2 cells. Data are representative images of Edu labelling. Scale bar, 18.75 μm. (**e**,**f**) A p62^mut^ expression vector that lacks the C-terminal LIR and UBA domains was constructed. HepG2 cells were trasfected with p62-siRNA alone or together with p62^mut^ expression vector. After 48 h of trasfection, cells were treated with Pep2–A2 or Pep2-con (5 μM) for 24 h, and then cell proliferation and invasion activities were evaluated through Edu (**e**) and transwell (**f**) assays. Scale bar, 18.75 μm (**e**) and 500 μm (**f**). (**g**,**h**) HepG2 cells were treated with Pep2-con, Pep2–A2 or Pep2–A2^mut^ (5 μM) for 24 h. The proliferation and invasion activities were evaluated with Edu (**g**) or transwell (**h**) assay. For all panels, *n*=3 independent experiments. Scale bar, 18.75 μm (**g**) and 500 μm (**h**). Data indicate mean±s.e.m. Statistical significance was determined with one-way ANOVA; **P<0.01; ***P<0.001.

To verify whether A2 interrupts the TRB3/p62 interaction in tumour cells, a fused peptide Pep2–A2 was designed by linking a cell-penetrating peptide^34^ to A2 using a glycine–glycine linker. Treatment of HepG2 cells with Pep2–A2 inhibited the TRB3/p62 interaction (Fig. 7a) and rescued the TRB3-reduced association of P62 with LC3 and ubiquitinated proteins (Fig. 7b,c). Thus, Pep2–A2 inhibited the colocalization of TRB3 and p62 with the reduction of TRB3 and p62 (Fig. 7d). Moreover, Pep2–A2^mut^ peptide carrying three, four, seven and nine alanine substitutions lost the binding capability with TRB3 (Fig. 6f) and showed less interfering effect on the p62/TRB3 interaction (Fig. 7e). It had reported that interactions of p62 with NBR1, RIP1, TRAF6 and Keap1 can regulate a diversity of cellular activities (Supplementary Fig. 6a)^24^,^35^. We found that Pep2–A2 did not interrupt the interactions of p62 with NBR1 or RIP1 but enhanced the interactions of p62/TRAF6 and p62/Keap1 in HepG2 cells (Supplementary Fig. 6b–e). In addition, Pep2–A2 did not affect the interaction of TRB3 with AKT and ATF4, two reported binding partners of TRB3 (Supplementary Fig. 6f). Interestingly, treatment of cancer cells with Pep2–A2 activated autophagic signalling and flux (Fig. 7f,g and Supplementary Movies 7–9), suggesting that Pep2–A2 is an autophagy-inducing peptide through interrupting the TRB3/P62 interaction. Moreover, Pep2–A2 activated UPS in these cells (Fig. 7h). We therefore explored whether interrupting this interaction produced anticancer actions. We found that Pep2–A2 decreased the expression of several tumour-promoting factors (Fig. 7i), which could be reversed by bafilomycin or MG132 (Fig. 7j), suggesting that Pep2–A2 reduces expression of the tumour-promoting factors by activating both autophagy and UPS. In addition, Pep2–A2 inhibited proliferation, migration and invasion in basal and IGF-1-stimulated HepG2 cells (Fig. 8a–d). The similar antitumour roles were observed in the Pep2–A2-treated A549 and HCT-8 cells (Supplementary Fig. 6g). To further confirm whether it is the p62/TRB3 interaction responsible for tumour-promoting effects of TRB3, we constructed a p62^mut^ expression vector that is p62-siRNA-resistant. This mutant lacks the C-terminal LIR and UBA domains and cannot interact with TRB3, but retains the self-oligomerization action. We found that the Pep2–A2 peptide showed no inhibiting effects on the proliferation and invasion of HepG2 cells with endogenous p62 depletion and isotopic expression of p62^mut^ (Fig. 8e,f). Moreover, Pep2–A2^mut^ peptide could not suppress tumour growth and invasion (Fig. 8g,h). These data demonstrate that the p62/TRB3 interaction is responsible for the tumour-promoting effects of TRB3.

**Figure 9:**
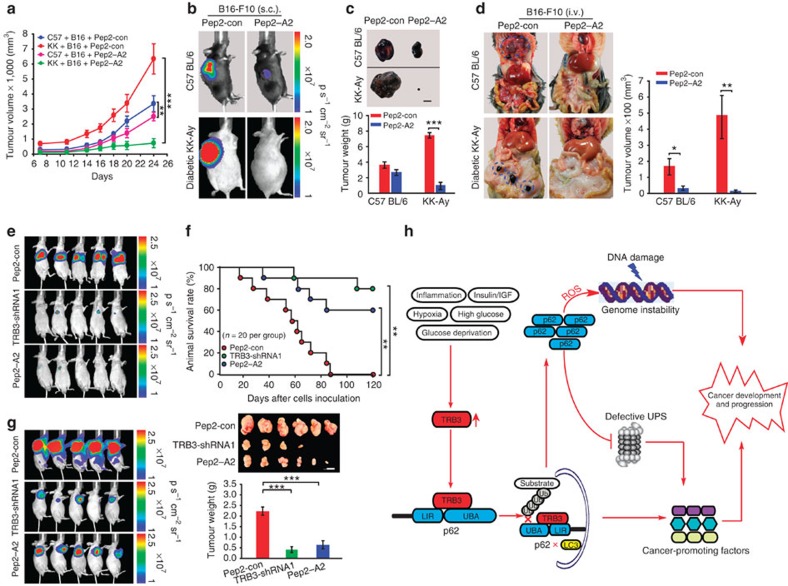
Interrupting the TRB3/p62 interaction inhibits tumour development and progression. (**a**–**c**) Pep2–A2 treatment inhibits tumour growth. KK-Ay and C57 BL/6 mice were s.c. inoculated with B16-F10 cells (1.5 × 10^5^). The animals were treated with Pep2–A2 or Pep2-con (5 mg kg^−1^) for indicated times. Data are tumour growth curves with mean volumes±s.e.m. at indicated times (**a**) representative graphs of mice (**b**) and tumours/quantified tumour weight (**c**; *n*=8 per group). Scale bar, 1 cm. (**d**) Pep2–A2 treatment inhibits metastasis. KK-Ay and C57 BL/6 mice were i.v. injected with B16-F10 cells (3 × 10^5^) and treated with Pep2-con or Pep2–A2 (*n*=11 per group). Data are representative graphs of animals (left) and total tumour volumes (mean±s.e.m.) at multiple metastatic sites (right). (**e**–**g**) Pep2–A2 treatment induces a similar antitumour efficacy with TRB3 silence. BALB/c nude mice were i.v. (3 × 10^6^) or s.c. (1 × 10^6^) injected with HepG2 cells or HepG2 cells expressing TRB3-shRNA1. One week later, the mice injected with the HepG2 cells were treated with Pep2-con or Pep2–A2 (5 mg kg^−1^) twice a week for 5 weeks. Data are representative of bioluminescence imaging (*n*=8 per group) (**e**) Kaplan–Meier survival curves for indicated groups of mice (*n*=20 per group; statistical significance determined with Kaplan–Meier log-rank test) and (**f**) photographs of representative mice (*n*=8 per group). Scale bar, 1 cm. (**g**). Statistical significance was determined with Student's *t*-test; *P<0.05, **P<0.01, ***P<0.001. (**h**) Schematic diagram illustrates the role of TRB3 in metabolic stresses induced cancer development and progression.

Indeed, the Pep2–A2 treatment attenuated tumour growth and metastasis in C57 BL/6 and KK-Ay mice inoculated with B16-F10 cells (Fig. 9a–d and Supplementary Table 5). We compared the antitumour role of TRB3 silencing or Pep2–A2 by using the nude mice i.v. injected with TRB3-silenced HepG2 cells or nude mice injected with control-shRNA HepG2 cells that were treated with Pep2–A2. We found that Pep2–A2 could attenuate pulmonary metastasis (Fig. 9e) and increase animal survival (Fig. 9f). In addition, Pep2–A2 inhibited tumour growth and decreased tumour sizes (Fig. 9g). Similar results were observed in nude mice inoculated with HCT-8 cells (Supplementary Fig. 7a,b). Finally, we examined whether Pep2–A2 inhibited primary recurrence and multiorgan metastasis after surgical resection of xenograft tumours. We found that Pep2–A2 as well TRB3 depletion inhibited tumour recurrence at primary location and multiple-organ metastasis (Supplementary Fig. 7c). Taken together, these results verify that interrupting the TRB3/p62 interaction produces a potent antitumour efficacy via restoring the p62 functions to speed the autophagy and UPS degradation (Fig. 9h).

## Discussion

Despite the fact that insulin and IGF-1 have been long assumed as a biological connection between energy-metabolic disorders and cancers, the defined mechanism remains incompletely understood^9^. The activation of insulin/IGF receptors triggers signalling cascades of the PI3K-AKT and MAPK/ERK pathways that promote mitogenesis and antagonize apoptosis in tumour development. Additional biological activities such as pro-angiogenesis, pro-migration and pro-glycolysis may also account for the insulin/IGF-induced tumour development^36^. However, there is controversy regarding the pro-mitogenic effects as the mechanisms of insulin/IGF-1 in promotion of cancer development. For instance, Weinberg points out recently that the primary role of insulin/IGF-1 is not to turn on the Warburg effect or to promote proliferation but is to suppress cell-suicide mechanisms^9^. In fact, clinical trials show that targeting insulin/IGF-1 signal does not produce satisfactory efficacy against cancers^7^. In this study, we demonstrate that metabolic stresses including insulin/IGF-1 enhance expression of TRB3 in a diversity of human tumour tissue and cells. TRB3 mediates insulin/IGF-induced reactive oxygen species production, DNA damage, increase in S-phase fraction, evasion of apoptosis and proliferation in cancer cells, whereas silencing TRB3 impedes the malignancy-promoting actions. TRB3 interacts with autophagic receptor p62 and interferes with the p62 cargo function and autophagic clearance of ubiquitinated proteins. Moreover, the p62 accumulation and dysfunction of selective autophagy by TRB3 inhibit UPS-dependent substrate degradation^29^. Defective autophagy and UPS result in accumulation of cancer-promoting factors including EGFR, COX2, MMP1/2, MT-MMP, c-Myc as well as Snail and Twist that play critical roles in regulation of tumour metabolism, proliferation, the epithelial-mesenchymal transition (EMT), invasion and autophagy^37^,^38^,^39^,^40^,^41^. Thus, TRB3 depletion suppresses tumour initiation, growth and metastasis in mice, particularly in mice with T2D. Our study establishes the stress protein TRB3 as a molecular link connecting insulin/IGF to cancer promotions (Fig. 9h).

Our studies demonstrate that the enhanced TRB3 represses the autophagic degradation as well as UPS substrate clearance, whereas silencing TRB3 induces autophagy and UPS in cancer cells. How a single stress protein plays such critical roles? First, TRB3 interacting with p62 is a key that causes p62 accumulation and p62-mediated autophagy dysfunction. Because TRB3 interferes with the binding of p62 LIR motif to LC3 and p62 UBA domain to ubiquitinated substrates, once the p62-binding sites are occupied by TRB3, loss of the p62 functions occurs so that the p62-binding substrates and p62 itself cannot be degraded and accumulate in cells. Indeed, the turnover of p62 is inhibited by a later-phase inhibitor of autophagy, verifying that TRB3 inhibits autophagic flux and degradation in cancer cells. In addition, sustained EGFR stability plays a critical role in the suppression of autophagy caused by the TRB3/p62 accumulation and interaction because the activation of EGFR suppresses autophagy as reported in ref. 41. Thus, silencing EGFR enhances autophagic flux and reduces p62 accumulation. Importantly, silencing TRB3 activates UPS by reducing p62 accumulation and restoring autophagic flux. These findings coincide with a report that p62 accumulation caused by autophagy inhibition slows down the clearance of UPS^29^. Our study suggests that a negative crosstalk between autophagy and UPS, the two key degradation systems in cells, is established in response to p62 accumulation and autophagy suppression caused by TRB3 enhancement (Fig. 9h). Of course, other mechanisms and molecular pathways may also contribute to the tumour-promoting effect of TRB3, such as the TGF-β/Smad3 and the Notch1 signalling pathways^13^,^42^,^43^.

The discovery of the critical roles of cargo receptor p62 in cancers provides a strong rationale for p62-based cancer therapy^44^. However, it seems a challenge for selectively inhibiting or eliminating p62 from cancer cells without interfering with p62 function in normal cells. In fact, p62 depletion does not produce an equivalent effect on expression of the tumour-promoting factors as well as antitumour action as TRB3 depletion. Silencing p62 may still show detrimental effects in response to IGF because IGF may induce TRB3 to bind with p62 and hinder the cargo function of residue p62 and autophagic degradation. In our study, depletion of p62 even results in the accumulation of several tumour-promoting factors, including MMP1, MT-MMP and Twist (Fig. 4g). Because of its double sword effect, the comprehensive results of p62 depletion cannot recapitulate the effects of TRB3 depletion. In contrast, TRB3 is a stress-induced protein that is highly expressed in human cancer or stressed cells but not in normal cells^10^. Therefore, silencing TRB3 endorses autophagy and UPS by interrupting the TRB3/p62 interaction and restoring the p62 cargo functions in cancer cells, which may provide an accessible targeted therapy approach for patients with T2D- or obesity-related cancers.

Targeting protein–protein interaction is emerging as a promising anticancer strategy^33^,^45^. Several PPI modulators that inhibit the interactions of MDM2/p53, XIAP/caspase-9 and BCL2/beclin1 are being tested in clinical trials for cancer patients^32^,^46^. In this study, we found that Pep2–A2 inhibits the interaction of TRB3/p62 and displays significant antitumour effects. We also found that the Pep2–A2 treatment enhanced the interactions of p62/TRAF6 and p62/Keap1 in HepG2 cells (Supplementary Fig. 5d,e). It was reported that the interaction of TRAF6 and p62 is important for cancer cell proliferation through the activation of mTORC1 (ref. 47). However, the p62/Keap1 interaction induces tumour inhibition through increasing the stability of Nrf2 (ref. 48). Thus, the therapeutic efficacy of Pep2–A2 against tumours may result from the comprehensive effects of the peptide on several protein–protein interactions. More studies should be carried out to reveal the exact mechanisms of pep–A2 for its antitumour effects.

In conclusion, our studies demonstrate that stress protein TRB3 mediates metabolic factors including insulin/IGF-induced tumour development and progression; this work provides the proof-of-concepts for targeting the TRB3/p62 interaction as a therapeutic strategy against cancers, particularly in T2D patients with cancers.

## Methods

### Plasmid construction

An Ub^G76V^ sequence was synthesized commercially and constructed in pEGFP-N1 vector (Clontech) to establish the Ub^G76V^-GFP-expressing plasmid. DYKDDDDK (DDK)-tagged p62 and its truncations, M1 (amino acids 1–102), M2 (amino acids 103–440), M3 (amino acids 103–343), M4 (amino acids 103–440, Δ321–390) and M5 (amino acids 103–320), were constructed into pCMV6-Entry (Origene) vector by standard subcloning.

### Semiquantitative reverse-transcription PCR and RNA interference

Total RNA was extracted using TRIzol (Invitrogen) following the manufacturer's instructions. Reverse transcription of the total cellular RNA was carried out using oligo(dT) primers and M-MLV reverse transcriptase (Promega). PCR was performed using an Mycycler thermal cycler and analysed using agarose gels. The sequences of the PCR primers were as follows: *TRB3* forward, 5′-AAGCGGTTGGAGTTGGATG-3′; *TRB3* reverse, 5′-GTCAGCGAAGACAAA GCGAC-3′; *p62* forward, 5′-TCGCTATGGCGTCGCTC-3′; *p62* reverse, 5′-CA CCCGAAGTGTCCGTGTTT-3′; *Actin* forward, 5′-GTGGACATCCGCAAAGACC-3′; *Actin* reverse, 5′-CCTAGAAGCATTTGCGGTG-3′. Full scans of agarose gels are presented in Supplementary Fig. 10.

p62 siRNAs were produced using RIBOBIO (Beijing, China) and transfected using Lipofectamine RNA interferenceMAX Transfection Reagent (Life Technologies) according to the manufacturer's instructions. Target sequences for p62 are p62-siRNA1 and p62-siRNA2.

### Cell culture and generation of stable cell lines

All the cell lines were provided from the Cell Culture Center of Peking Union Medical College or Chinese Academy of Sciences, where they were recently authenticated by STR profiling, and characterized by mycoplasma detection and cell vitality detection. Cell lines were cultured under standard conditions. To generate cell populations stably expressing TRB3-shRNA, the control-shRNA or the TRB3-shRNA plasmids were transfected into HepG2 or HCT-8 cells with siPORT XP-1 tansfection reagent (Ambion) according to the manufacturer's instructions. After 24 h of transfection, stable transfectants were selected in medium containing 200 mg ml^−1^ hygromycin (Calbiochem, San Diego, CA, USA) for 7 days. After two or three passages in the presence of hygromycin, the cultures were used for experiments without cloning. For bioluminescent tracking, cells were lentivirally infected with a reporter construct encoding green fluorescent protein and firefly luciferase. GFP-positive cells were enriched by fluorescence-activated cell sorting.

### Human cancer specimens and tissue microarray

Human primary liver, colon and lung carcinomas, and normal liver, colon and lung tissue specimens surgically removed and snap-frozen in liquid nitrogen were obtained from Alenabio (Xian, China) along with pathologic information (Supplementary Table 4). Protein extracts were prepared from three normal tissues and five tumour tissues. Frozen human cancer and adjacent non-cancer tissue microarrays were purchased from US Biomax (FLV402 for HCC, FCO405a for colon cancer and FLC320 for lung cancer). Paired cancer and adjacent non-cancer paraffin tissue sections (HLiv-HCC180Sur-02 for HCC, HCol-Ade180Sur-03 for colon cancer and HLug-Squ150Sur-01 for lung cancer) were purchased from Shanghai Outdo Biotech (Shanghai, China). All protocols using human specimens were approved by the Institutional Review Board of Chinese Academy of Medical Sciences and Peking Union Medical College. Informed consent was obtained from all subjects. The study conforms to the principles outlined in the Declaration of Helsinki.

### Animal studies

Athymic BALB/c nude mice (5–6-week old, male) and C57 BL/6 (male) mice were purchased from Vital River Lab Animal Technology Co., Ltd (Beijing, China). KK-Ay (male) mice were purchased from Beijing HFK Bioscience Co., Ltd (Beijing, China). All animals were maintained in animal facility at the Institute of Materia Medica under Specific Pathogen Free (SPF) conditions. KK-Ay mice were fed with high-fat diet; blood glucose was detected weekly. The blood glucose level of KK-Ay mice reached to 16 mM at 6 weeks of age and were considered to be diabetic mice used in the studies of Figs 2 and 9 and Supplementary Figs 2 and 4. For animal studies, the mice were earmarked before grouping and then were randomly separated into groups by an independent person; however, no particular method of randomization was used. Sample size was predetermined empirically according to previous experience using the same strains and treatments. Generally, we used *n*>6 mice per genotype and condition. We ensured that experimental groups were balanced in terms of animal age and weight. All animal procedures were conducted in accordance with the guidelines of the Institutional Committee for the Ethics of Animal Care and Treatment in Biomedical Research of Chinese Academy of Medical Sciences and Peking Union Medical College (Permit No. 002802).

### Mouse models for tumour growth and metastasis

To generate mouse models of tumour growth, 1.5 × 10^6^ HepG2 or HCT-8 cells per mouse in 200 μl PBS were s.c. injected into the right flank of 5-week-old athymic BALB/c nude mice or 1.5 × 10^5^ B16-F10 cells per mouse in 200 μl PBS were s.c. injected into the right flank of KK-Ay and C57 BL/6 mice. Tumour growth was monitored externally using vernier calipers on the indicated days. Tumour volume (*T*_V_) was calculated by the formula (*T*_V_=*W*^2^ × *L* × 0.5)^49^. For experimental metastasis, 5–6-week-old male athymic BALB/c nude, KK-Ay or C57 BL/6 mice were used as transplant recipients. Tumour cells were injected into the lateral tail vein (for athymic BALB/c nude mice, 3 × 10^6^ HepG2 or HCT-8 cells per mouse in 200 μl PBS; for KK-Ay or C57 BL/6 mice, 3 × 10^5^ B16-F10 cells per mouse in 200 μl PBS). Metastases were counted in a genotype-blinded manner under dissection scope. Tumour growth and tumour metastasis were also quantified using *in vivo* imaging system.

### MG132 and 3-MA treatment *in vivo*

Seven-week-old KK-Ay mice were i.p. injected with TRB3-shRNA lentiviral particles (2 × 10^5^ plaque-forming unit) to generate KK-Ay/TRB3-KD mice; over 75% depletion of TRB3 in target tissues (lungs and liver) was considered as effectively knocking down. 3-MA (30 mg kg^−1^ per day) or MG132 (2.5 mg kg^−1^ twice a week) was i.p. injected to the KK-Ay/TRB3-KD mice, from day 9 after tumour inoculation to the time of being killed. The evaluation of tumour growth and metastasis was described in the section of ‘Mouse Models for Tumour Growth and Metastasis'.

### Mouse models for tumorigenesis

To generate DEN-induced carcinogenesis model, C57 BL/6 mice were infected i.p. with 1 × 10^8^ viral particles of TRB3 adenovirus or GFP adenovirus per mouse on weeks 2, 3, 4, 5, 6, 7, 8 and 9 after birth. All mice were i.p. injected with DEN (25 mg kg^−1^; Sigma-Aldrich, St Louis, MO) at 4 weeks of age. Mice were killed at week 41 to observe tumour development. To generate DMBA-induced carcinogenesis model, DMBA was dissolved in sesame oil to give a 10-mg ml^−1^ stock concentration. Male KK-Ay and C57 BL/6 mice (5-week old) were gavaged per oral (p.o.) with 0.1 ml (total 1 mg) DMBA once a week for 5 weeks. Mice were killed at the age of 15 weeks or either if they displayed significant weight loss and signs of distress.

### Mouse models for spontaneous metastasis

For spontaneous metastasis assays, tumour-bearing mice (with tumour cells injected s.c. into the right flank) were anaesthetized and tumours were resected when tumour volumes reached 300–400 mm^3^ (4–5 weeks after cell injection)^50^. After tumour resection, the skin was closed with stainless steel clips.

### Western blot and immunostaining

Cell or tissue extracts were prepared with RIPA lysis buffer (Cell Signaling Technology). Lysates were resolved using SDS–PAGE and transferred to polyvinylidene difluoride members for immunoblotting. For insoluble fraction preparation, pellets were washed four times with RIPA and resuspended in 2% SDS in Tris-buffered saline (50 mM Tris, 150 mM NaCl, pH 7.4).

For immunofluorescence staining, cells grown on coverslips were fixed with 4% buffered paraformaldehyde. Human cancer tissue microarray (US Biomax) was rinsed twice with PBS. Then, cells were permeabilized with 0.5% Triton X-100 for 15 min. The slides were incubated with indicated antibodies at 4 °C overnight. After being washed with PBS for three times, cells were stained with goat anti-mouse Alexa 488 (1:500) and/or goat anti-rabbit Alexa 647 (1:500) secondary antibodies for 30 min at 37 °C, and then washed three times with PBS and mounted on glass slides. Nuclei were visualized with 4, 6-diamidino-2-phenylindole staining. Images were acquired using a confocal microscope (Leica Microsystems, Heidelberg, GmbH, TCS SP2).

Immunohistochemical staining was performed using commercial human cancer tissue microarray slides (Shanghai Outdo Biotech) with 3,3-diaminobenzidine. The sections were scanned at × 200 magnification. The images were then digitalized, and the integrated optical density (IOD) of TRB3 or pIRS-1 were calculated using software Image-Pro plus 5.1. The antibodies used are listed in Supplementary Table 6. Full scans of immunoblots are presented in Supplementary Figs 8–23.

### Immunoprecipitation and GST pull-down

For co-immunoprecipitation, cells were grown in ϕ10-cm dishes. Total cell lysate (5 mg protein) was subjected to immunoprecipitation with indicated antibodies overnight at 4 °C with gentle agitation, followed by incubation with Protein A/G Plus-Agarose for 2∼4 h at 4 °C. The immunocomplex was washed three times and mixed with 2 × SDS sample buffer and boiled for 5 min. The co-precipitates were resolved using SDS–PAGE and detected with immunoblotting. For GST pull-down assays, p62-GST or TRB3-GST plasmid was transformed into *Escherichia coli* strain BL21. Recombinant protein expression was induced with 0.2 mM isopropyl-b-D-thiogalactoside at 16 °C overnight. The purified and immobilized GST fusion proteins were incubated with His-TRB3/His-p62-purified protein. After washing, bound proteins were analysed by immunoblotting with anti-His antibody.

### Migration and invasion assays

Confluent cell monolayers were wounded by manually scraping the cells with a pipette tip, washed with PBS and further cultured in DMED medium supplemented with 0.4% fetal bovine serum (FBS) for 48 h. Images were captured at 0 and 48 h after wounding with an Olympus CKX41 microscope, and the lesion area was measured (medium alone containing 0.4% FBS were used as control).

Transwell invasion assays were performed using Transwell chambers with filter membranes of 8-μm pore size (Millipore). Chambers were precoated with 10 μg ml^−1^ Fibronectin on the lower surface, and the polycarbonate filter was coated with Matrigel (30 μg per well; BD Matrigel Matrix). Then, the chambers were inserted in 24-well culture plates. Cells were starved overnight in assay media (DMEM media containing 0.4% FBS), and then single-cell suspensions were seeded into the upper chamber (5 × 10^4^ cells per well in 0.4% FBS in DMEM). After 24 h, non-invaded cells on the upper side of the filter were removed with a cotton swab. Cells invaded were fixed with 4% paraformaldehyde in PBS, stained with 0.5% toluidine in 2% Na_2_CO_3_ and counted using brightfield microscopy at × 200 in eight random fields.

For three-dimensional (3D) Matrigel culture^51^, single cells (2 × 10^3^ per well performed in triplicate) were mixed into 0.4 ml of DMEM medium supplemented with 10% FBS and 5% chilled growth factor-reduced Matrigel (BD Biosciences), and cultured in suspension in 24-well ultra-low attachment plate (Corning) at 37 °C for 6–10 days. Matrigel was replenished every 3 days. Phase-contrast images of the 3D structure were taken on day 8.

### Live-cell imaging for autophagic flux

The mRFP-GFP-LC3 adenoviral particles were purchased from HanBio (Shanghai, China). Cells were infected with adenoviral particles; after infection, the cells were cultured for another 24 h. Imaging was performed on a UltraVIEW VoX 3D Live Cell Imaging System^52^. All image acquisition settings were kept at the same state during the image collection. Image analysis was performed using the Volocity Demo 5.4 software. For collecting images to prepare movies, images were taken at a 5-s interval, and then processed and merged using the Volocity Demo 5.4 software and exported as .avi files with four frames per second.

### Transmission electron microscopy

For TEM, cells were immediately fixed with 2.0% glutaraldehyde in 0.1 M sodium cacodylate buffer, pH 7.4, and then post-fixed in 1% osmium tetroxide, dehydrated in ethanol and embedded in epon. Ultrathin sections of HepG2 cells expressing control-shRNA, TRB3-shRNA1 and TRB3-shRNA2 were collected on formvar-coated grids and were stained with uranil acetate and lead citrate. The samples were examined with a HITACHI H600 Transmission Electron Microscope operated at 80 KV.

### Surface plasmon resonance analysis

Binding kinetics between TRB3 and control, P2, P4, P6, P7, P8, P9, P11, A1, A2 or A3 were measured by a surface plasmon resonance assay using BIAcore T200 instrument (GE Healthcare, Pittsburgh, USA)^53^. The dissociation constant (*K*_D_) was calculated according to the BIA-evaluation software.

### Peptide competition assay

Ninety-six-well plates were coated with the target protein TRB3 or BSA (overnight) and washed. After incubation with p62 protein or HRP-conjugated peptides (1 h), the wells were washed and peptide TRB3 or p62–TRB3 binding was measured at 450 nm after reaction with HRP substrates. For competitive enzyme-linked immunosorbent assay (ELISA), p62 binding to TRB3 was competed with 0.1–100 μg ml^−1^ (1 h) of HRP-non-conjugated A2 or control^54^. For immunoprecipitation assay, control or Pep2–A2 peptides (5 μM) were added to HepG2 cells for 12 h. Then, cell lysates were collected for immunoprecipitation as described above.

### Peptide treatment

To determine the antimetastatic effect of peptide, mice (5-week old) were tail-vein-injected with the indicated types of cancer cells (HepG2 or HCT-8 1.5 × 10^6^ each mouse, B16-F10 1.5 × 10^5^ each mouse). One week later, the mice were i.v. treated with the Pep2–A2 (5 mg kg^−1^) twice a week for 5 weeks. For survival rate evaluation, nude mice were tail-vein-injected with HepG2 cells expressing control-shRNA or TRB3-shRNA1. One week after tumour inoculation, control-shRNA mice were treated with the Pep2–A2 or Pep2-Con (5 mg kg^−1^) twice a week for 4 months. To measure the tumour-growth-inhibiting effect of the peptide, mice were injected with cancer cells into the right flank. One week after tumour inoculation, mice were i.v. treated with the Pep2–A2 (5 mg kg^−1^) twice a week for 5 weeks. For spontaneous metastasis assays, the treatment group received i.v. injections of Pep2–A2 (5 mg kg^−1^) twice a week for 2 months on day 1 after surgery. Tumour growth, metastasis and recurrences at the primary site and multiorgan metastasis were quantified using *in vivo* imaging system. To measure the prevention effect of peptide on tumorigenesis, C57 BL/6 and KK-Ay mice were i.v. injected with the Pep2–A2 (5 mg kg^−1^) twice a week from 4 weeks of age till the time of killing at 15 weeks of age. DMBA was dissolved in sesame oil to give a 10-mg ml^−1^ stock concentration and mice were gavaged p.o. with 0.1 ml (total 1 mg) DMBA once a week at 5 weeks of age for 5 weeks.

To determine the roles of autophagy in the therapeutic effect of Pep2–A2 on tumour progression, 3-MA, an inhibitor of autophagy, was given to tumour growth and metastasis model of C57 BL/6 and KK-Ay mice. 3-MA (30 mg kg^−1^ per day) was i.p. injected to mice treated with Pep2-con or Pep2–A2, from day 9 after tumour inoculation to the time of being killed. To determine the roles of UPS in the therapeutic effect of Pep2–A2 on tumour progression, MG132, an inhibitor of proteasome, was given to tumour growth and metastasis model of C57 BL/6 and KK-Ay mice. MG132 (2.5 mg kg^−1^ twice a week) was i.p. injected to mice treated with Pep2-con or Pep2–A2 from day 9 after tumour inoculation to the time of being killed. The evaluation of tumour growth and metastasis was described in the section of ‘Mouse Models for Tumour Growth and Metastasis'.

### Bioluminescent imaging and analysis

Mice were anaesthetized and injected with 1.5 mg of D-luciferin (i.p., 15 mg ml^−1^ in PBS). Imaging was completed between 2 and 5 min after injection with a Xenogen IVIS system coupled to Living Image acquisition and analysis software (Xenogen). For bioluminescent imaging plots, the photon flux was calculated for each mouse by using a rectangular region of interest encompassing the thorax of the mouse in a prone position. This value was scaled to a comparable background value (from a luciferin-injected mouse with no tumour cells), and then normalized to the value obtained immediately after xenografting (day 0).

### Statistical analysis

Data were analysed by Student's *t-*test or one-way analysis of variance followed by Tukey's honestly significant difference (HSD) test using SPSS 13.0 (SPSS Inc.). Survival curves were analysed by Kaplan–Meier log-rank (Mentel–Cox) test. Sample size of most experiments was chosen empirically following previous experience in the assessment of experimental variability. Generally, all experiments were carried out with *n*≥3 biological replicates. *P*<0.05 was statistically significant.

## Additional information

**How to cite this article:** Hua, F. *et al.* TRB3 links insulin/IGF to tumour promotion by interacting with p62 and impeding autophagic/proteasomal degradations. *Nat. Commun.* 6:7951 doi: 10.1038/ncomms8951 (2015).

## Supplementary Material

Supplementary Information Supplementary Figures 1-23 and Supplementary Tables 1-6

Supplementary Movie 1 Autophagolysosomes (red) and autophagosomes (red and green) in Control-shRNA cells.

Supplementary Movie 2 Autophagolysosomes (red) and autophagosomes (red and green) in HepG2-shRNA1 cells.

Supplementary Movie 3 Autophagolysosomes (red) and autophagosomes (red and green) in HepG2-shRNA2 cells.

Supplementary Movie 4 Autophagolysosomes (red) and autophagosomes (red and green) in Control-shRNA cells treated with IGF-1 (100 nM) for 12 hr.

Supplementary Movie 5 Autophagolysosomes (red) and autophagosomes (red and green) in HepG2-shRNA1 cells treated with IGF-1 (100 nM) for 12 hr.

Supplementary Movie 6 Autophagolysosomes (red) and autophagosomes (red and green) in HepG2-shRNA2 cells treated with IGF-1 (100 nM) for 12 hr.

Supplementary Movie 7 Autophagolysosomes (red) and autophagosomes (red and green) in HepG2 cells.

Supplementary Movie 8 Autophagolysosomes (red) and autophagosomes (red and green) in HepG2 cells treated with Pep2-con for 12 hr.

Supplementary Movie 9 Autophagolysosomes (red) and autophagosomes (red and green) in HepG2 cells treated with Pep2-A2 for 12 hr.
