# Supplementary material for: TRB3 links insulin/IGF to tumour promotion by interacting with p62 and impeding autophagic/proteasomal degradations
Source: Nat Commun. 2015 Aug 13;6:7951. doi: 10.1038/ncomms8951 (PMC4557121; doi:10.1038/ncomms8951)
Supplement: Supplementary Information — Supplementary Figures 1-23 and Supplementary Tables 1-6 [file ncomms8951-s1.pdf]

Fig-S1 (Hu)

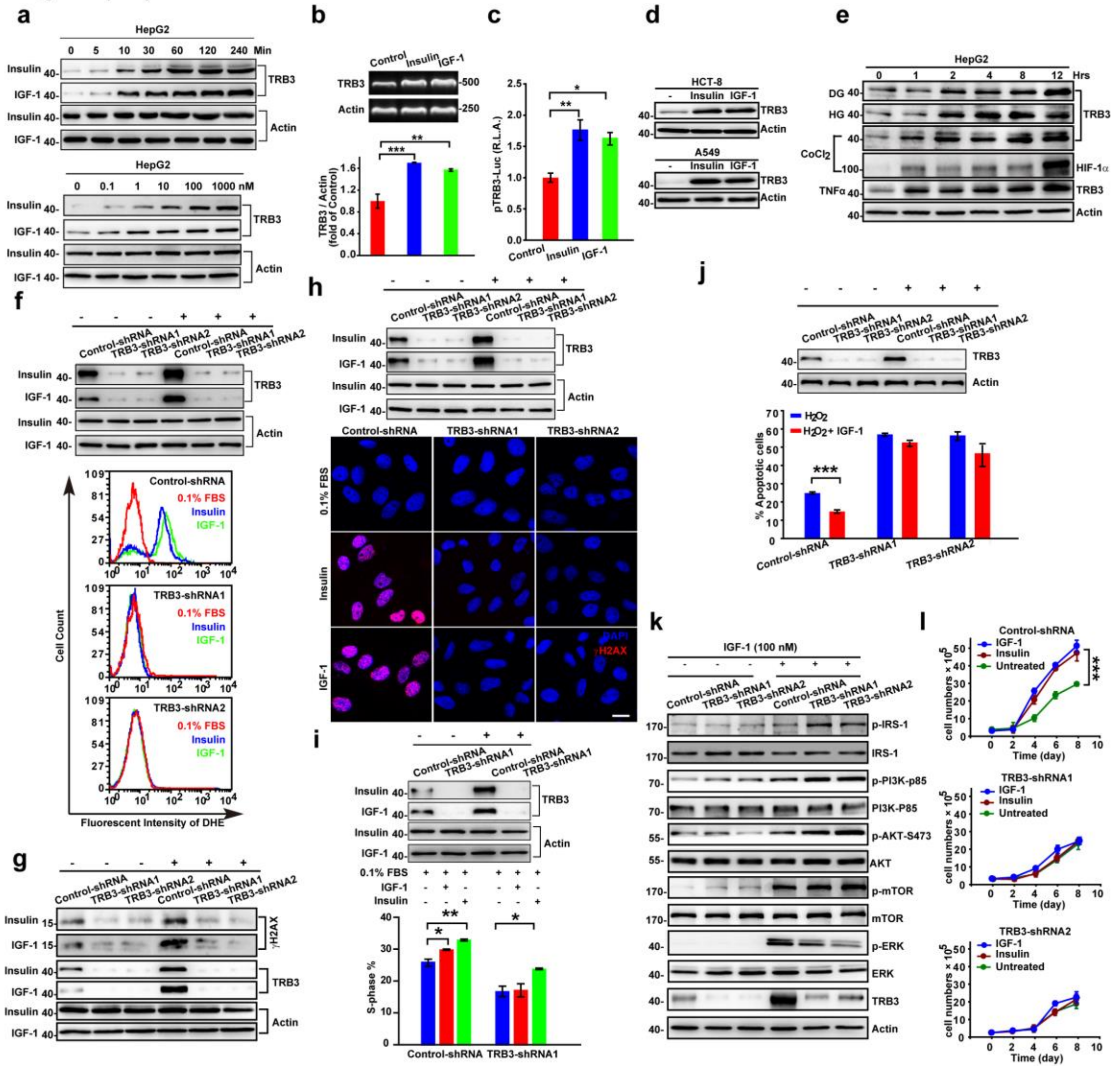


**Supplementary Figure 1. Metabolic stress induced TRB3 mediates the cancer-promoting roles.** (**a**) HepG2 cells were treated with insulin/IGF-1 (100 nM) for indicated times (upper) or treated with insulin/IGF-1 at different concentrations for 12 hours. The expression of TRB3 was detected with immunoblotting. Data are representative immunoblots of 5 independent assays. (**b**) HepG2 cells were treated with insulin (100 nM) or IGF-1 (100 nM) for 12 hours. RT-PCR analysis was carried out to determine the mRNA level of TRB3 and β-actin. Data are mean ± s.e.m. of 3 independent assays with triplicates. (**c**) HepG2 cells were transfected with pTRB3-luc plasmid for 24

hours. The cells were treated with insulin (100 nM) or IGF-1 (100 nM) for another 12 hours and the luciferase activity was measured. Data are mean ± s.e.m. of 5 independent assays with triplicates. (**d**) HCT-8 or A549 cells were treated with insulin (100 nM)/IGF-1 (100 nM) for 4 hours. The TRB3 levels were determined with immunoblot (*n*=5 independent assays). (**e**) HepG2 cells were treated with glucose deprivation (DG), high glucose (HG, 50 mM), $CoCl_2$ (200 μM) and TNFα (50 ng $ml^{-1}$) for indicated time points respectively. TRB3 levels were determined with immunoblot analysis (*n*=5 independent assays). (**f**) TRB3 depletion suppressed insulin/IGF-1-induced ROS generation. ROS was determined with Dihydroethidium (DHE, 10 μM) staining followed with flow cytometry (*n*=5 independent assays). (**g**,**h**) TRB3 depletion attenuated insulin/IGF-1-induced DNA damage. The expression of γH2AX was evaluated with immunoblotting (g) or immuno-fluorescence staining (h), scale bar, 18.75 μm, *n*=5 independent assays. (**i**) TRB3 depletion decreased the S-phase fraction and inhibited the insulin/IGF-induced prolongation of S-phase. Data are mean ± s.e.m. of 3 independent assays with triplicates. (**j**) TRB3 depletion increased sensitivity of cancer cells to hydrogen peroxide-induced apoptosis. Data are mean ± s.e.m. of 3 independent assays with triplicates. (**k**) HepG2 cells were treated with or without IGF-1 for 30 min and expressions of indicated proteins were detected with immunoblotting (*n*=5 independent assays). (**l**) Depletion of TRB3 inhibited insulin/IGF-1-induced proliferation of cancer cells. Data are mean ± s.e.m. of 3 independent assays with triplicates.

Statistical significance was determined by Student's *t*-test; *$P$<0.05; **$P$<0.01; ***$P$<0.001.

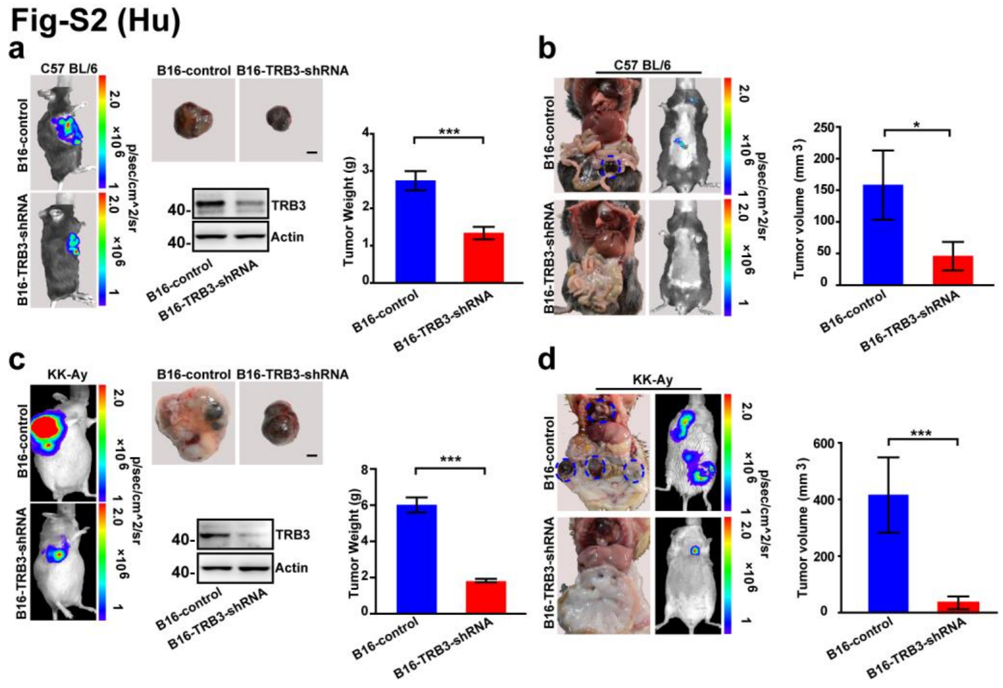


**Supplementary Figure 2.TRB3 depletionin tumor cells displays much prominent antitumour effects in diabetic KK-Ay mice than that in C57BL/6 mice.** (**a**) C57 BL/6 mice were *s.c.* inoculated with B16-F10 cells expressing control-shRNA or TRB3-shRNA ($1.5\times10^5$). Data are representative bioluminescence images and tumours along with quantified tumour weight. The levels of TRB3 were also shown in B16-F10 cells expressing control-shRNA or TRB3-shRNA. Scale bar, 1.5 cm, $n$=8 per group. (**b**) C57 BL/6 mice were *i.v.* injected with B16-F10 cells expressing control-shRNA or TRB3-shRNA ($3\times10^5$). Data are bioluminescence images with total tumour volumes at multiple metastatic sites (mean ± s.e.m.; $n$=8 per group). (**c**) KK-Ay T2D mice were *s.c.* inoculated with B16-F10 cells expressing control-shRNA or

TRB3-shRNA ($1.5\times10^5$). Data are representative bioluminescence images and tumours along with quantified tumour weight. The levels of TRB3 were also shown in B16-F10 cells expressing control-shRNA or TRB3-shRNA. Scale bar, 1.5 cm, *n*=8 per group. (**d**) KK-Ay T2D mice were *i.v.* injected with B16-F10 cells expressing control-shRNA or TRB3-shRNA ($3\times10^5$). Data are bioluminescence images with total tumour volumes at multiple metastatic sites (mean ± s.e.m.; *n*=8 per group). Statistical significance was determined with Student's *t*-test; **P*<0.05, ****P*<0.001.

Fig-S3 (Hu)

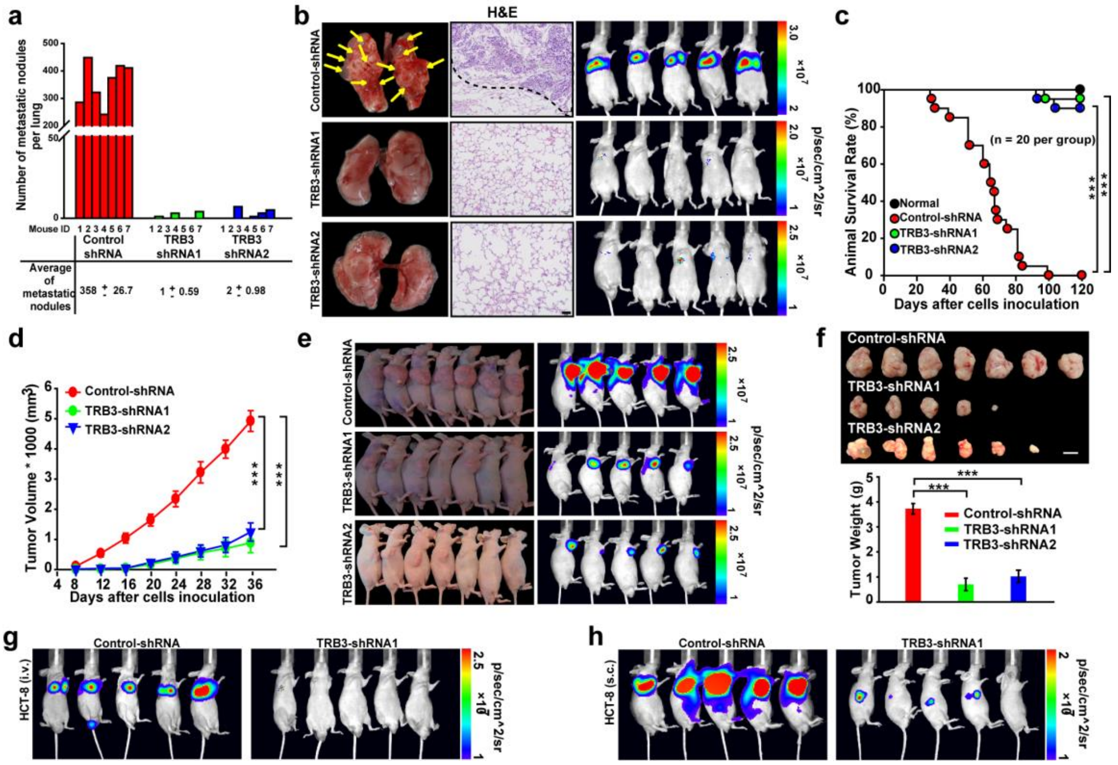


**Supplementary Figure 3. TRB3 depletion protects against tumour metastasis and growth.** (**a,b**) BALB/c nude mice were *i.v.* injected with HepG2 cells expressing control-shRNA, TRB3-shRNA1 or TRB3-shRNA2

($3\times10^6$). *In vivo* imaging system was used to detect tumour metastasis. Data are number of metastases in lungs (a). Representative graphs of lungs, H&E stained lung sections and bioluminescence imaging (b). Metastatic nodules were indicated by arrows. Scale bar, 5 μm, *n*=10 per group. (**c**) Kaplan-Meier survival curve for mice harboring tumours expressing control-shRNA or one of the two shRNAs targeting TRB3 (*n*=20 per group). Statistical significance was determined by Kaplan-Meier log-rank test; ****P*<0.001. (**d**) HepG2 cells expressing control-shRNA, TRB3-shRNA1 or TRB3-shRNA2 ($1.5\times10^6$) were *s.c.* injected into the right flank of the BALB/c nude mice. Data are mean volumes ± s.e.m. at indicated times and photographs of representative mice (*n*=10 per group). (**e,f**) Data are photographs of representative mice (e), and tumours along with quantified tumour weight (f). Scale bar, 1.5 cm, *n*=10 per group. (**g,h**) BALB/c nude mice were *i.v.* ($3\times10^6$) or *s.c.* ($1.5\times10^6$) injected with HCT-8 cells expressing control-shRNA or TRB3-shRNA1. *In vivo* imaging system was used to detect tumour metastasis and growth. Data are representative bio-photonic images of animals with metastasis (g) and growth (h) (*n*=6 per group). Statistical significance was determined by Student's *t*-test; ****P*<0.001.

Fig-S4 (Hu)

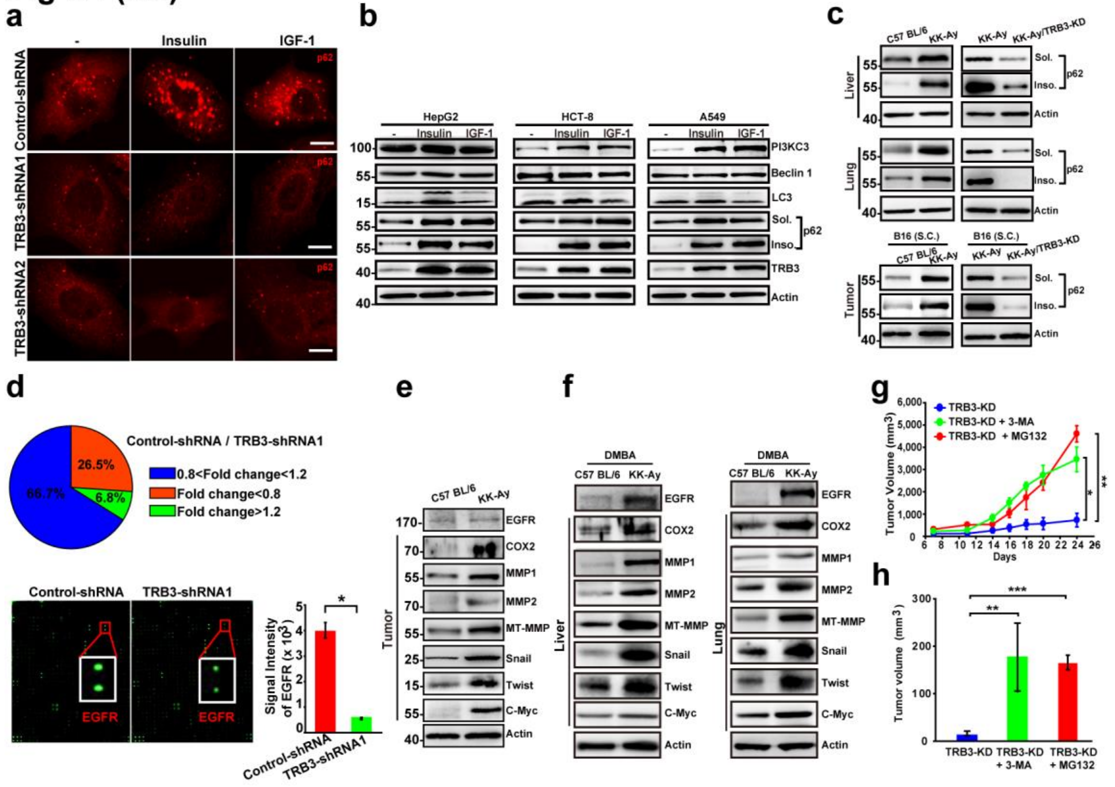


**Supplementary Figure 4. Metabolic stresses induce autophagy inhibition and tumour-promoting factor accumulation.** (**a**) HepG2 cells were treated with the indicated stimulators for 12 hr and p62 expression was evaluated with an indirect immunofluorescence (*n*=3 independent assays). Scale bar, 7.5 μm. (**b**) HepG2, HCT-8 and A549 cells were treated with or without insulin/IGF-1 (100 nM) for 12 hours. The expressions of autophagy related proteins were evaluated with immunoblotting (*n*=5 independent assays). (**c**) The expression of soluble and insoluble p62 in liver, lung tissues and xenograf ttumours were evaluated with immune-blotting in C57 BL/6, KK-Ay and KK-Ay/TRB3-KD mice. Data are representative immunoblots of 4 independent assays. (**d**) The cell-wide protein expression was evaluated with Human L-1000 Antibody Arrays in HepG2 cells expressing TRB3-shRNA1 or

control-shRNA. Data are the summarized fold changes of proteins (control-shRNA/TRB3-shRNA1) (upper) and representative spots showing differentially expressed EGFR (framed) (lower). Data are representative of 2 independent assays. (**e**) The xenograft tumours express higher pro-metastasis and pro-EMT factors in KK-Ay mice than that in C57 BL/6 mice. Data are representative immunoblots of 4 independent assays. (**f**) The liver and lung tissues express higher pro-metastasis and pro-EMT factors in DMBA-treated KK-Ay mice than that in DMBA-treated C57 BL/6. Data are representative immunoblots of 4 independent assays. (**g,h**) Suppressing autophagy or UPS reverses the antitumour effect of TRB3 knockdown. TRB3 knockdown KK-Ay mice were *s.c.* injected with B16-F10 cells ($1.5\times10^5$) or *i.v.* injected with B16-F10 cells ($3\times10^5$) after infection. The mice were treated with 3-MA (30 mg $kg^{-1}$ per day) or MG132 (2.5 mg $kg^{-1}$, twice a week) from day 7 after tumour inoculation. Data are mean ± s.e.m. of tumour growth curve (g) and total tumour volumes in multiple metastatic sites (h) ($n$=6 per group). Statistical significance was determined by Student's *t*-test; *$P$<0.05, **$P$<0.01, ***$P$<0.001.

Fig-S5 (Hu)

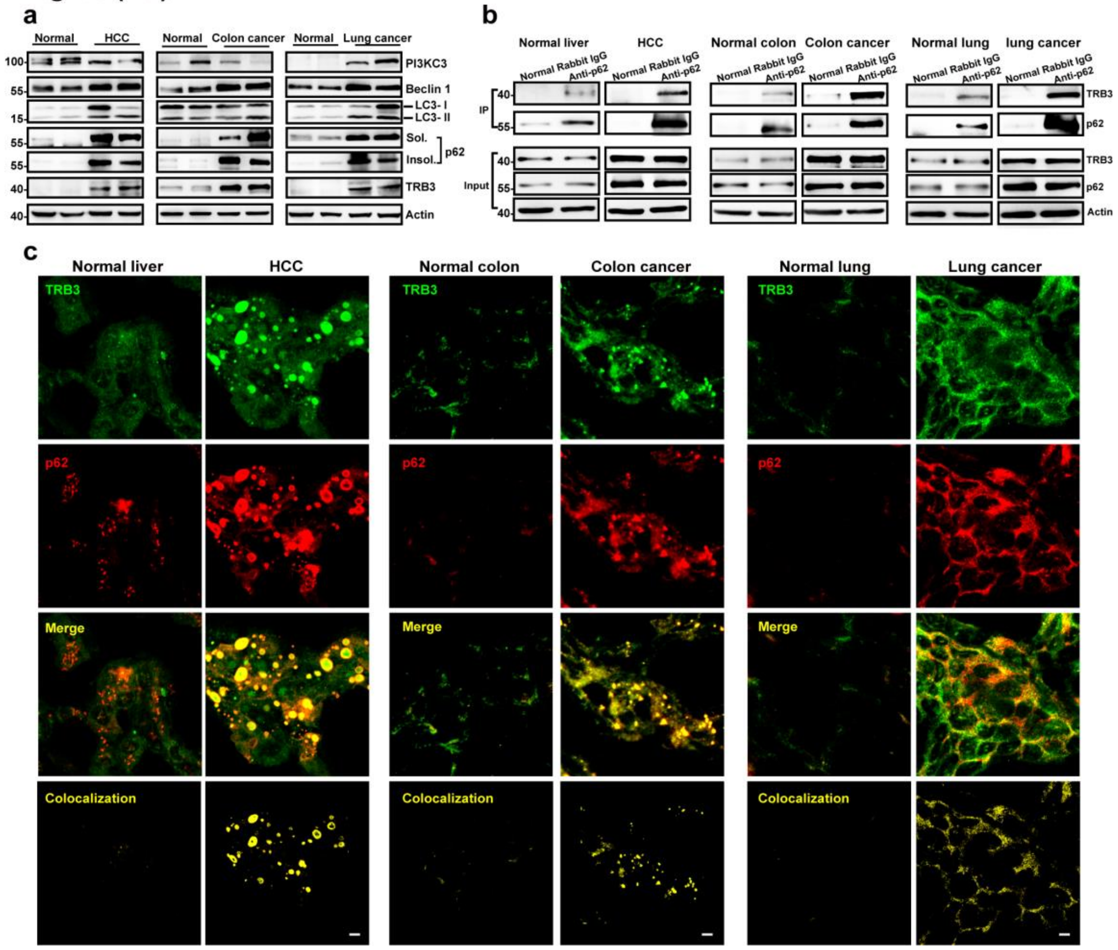


**Supplementary Figure 5. The interaction of TRB3/p62 is enhanced in human tumourtissues.** (**a**) Expressions of autophagy molecules in HCC, colon and lung cancer tissues were evaluated with immunoblotting (*n*=5 cases). Normal tissue extracts were used as control (*n*=3 cases). Data are representative immunoblots of 3 independent assays. (**b**) Co-IP of endogenous TRB3 and p62 proteins from fresh normal human (*n*=3 cases) and tumour tissue (*n*=5 cases) samples. Tissue extracts were IP with anti-p62 Ab or rabbit IgG and blotted with anti-TRB3 Ab. (**c**) Human normal and cancer tissue array slides were double immunostained with anti-TRB3 (green) or anti-p62 (red) Ab. Double staining in yellow shows TRB3/p62co-localization. Scale

bar, 5 μm. Each columnis representative of double-stain (*n*=10 cases).

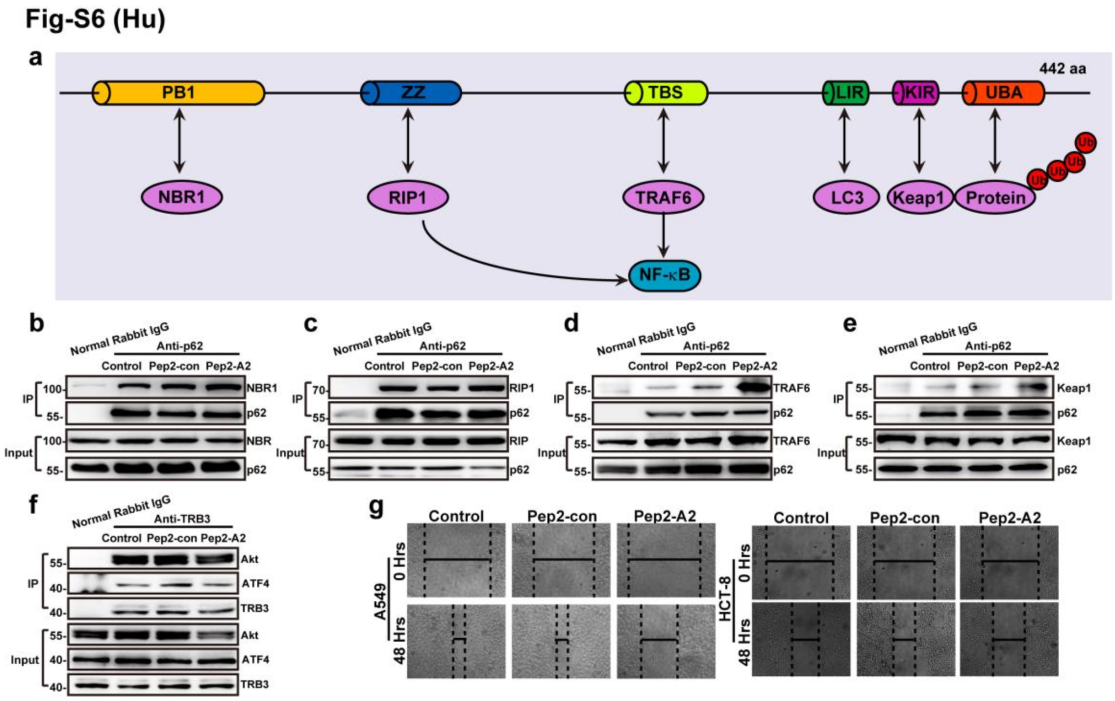


**Supplementary Figure 6. Pep2-A2 treatment increases the binding of p62 to TRAF6 and Keap1.** (**a**) Domain structure of p62. (**b-e**) The extracts of HepG2 cells treated with Pep2-con or Pep2-A2 were IP with an anti-p62 Ab or normal rabbit IgG and blotted with anti-NBR1, anti-RIP1, anti-TRAF6 or anti-Keap1 Ab. Data are representative immunoblots of 3 independent assays. (**f**) The extracts of HepG2 cells treated with Pep2-con or Pep2-A2 were IP with an anti-TRB3 Ab or normal rabbit IgG and blotted with anti-AKT or anti-ATF4 Ab. Data are representative immunoblots of 3 independent assays. (**g**) Wound healing assay was conducted in A549 and HCT-8 cells treated with 5 μM of Pep2-A2 or Pep2-con at the indicated times. Data are representative of 3 independent assays.

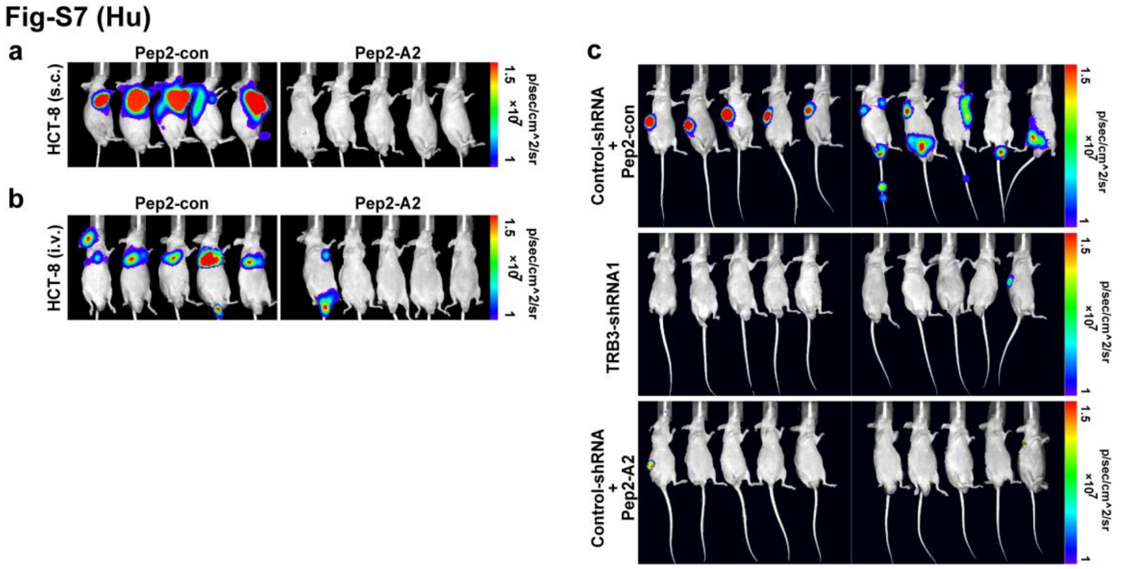


**Supplementary Figure 7. The Pep2-A2 treatment inhibits tumour development and progression.** (**a**, **b**) Nude mice were *s.c.* or *i.v.* inoculated with HCT-8 cells into the right flank ($1.5 \times 10^6$) or the lateral tail vein ($3 \times 10^6$). One week after tumour inoculation, mice were *i.v.* treated with the Pep2-A2 or Pep2-con (5 mg $kg^{-1}$) twice a week for 5 weeks. Data are representative bio-photonic images (*n*=6 per group). (**c**) Pep2-A2 inhibits primary recurrence and multi-organ metastasis. Nude mice were *s.c.* inoculated with HepG2 cells expressing control-shRNA1 (*n*=20) or TRB3-shRNA2 (*n*=10). When tumours reached a volume of 300 $mm^3$, tumour resection was performed. The control-shRNA mice (n=10) were randomly selected for treating with Pep2-A2 for 2 months (5 mg $kg^{-1}$, *i.v.*, beginning 1 day post-resection). Data are representative bio-photonic images of animals with primary tumour recurrence.

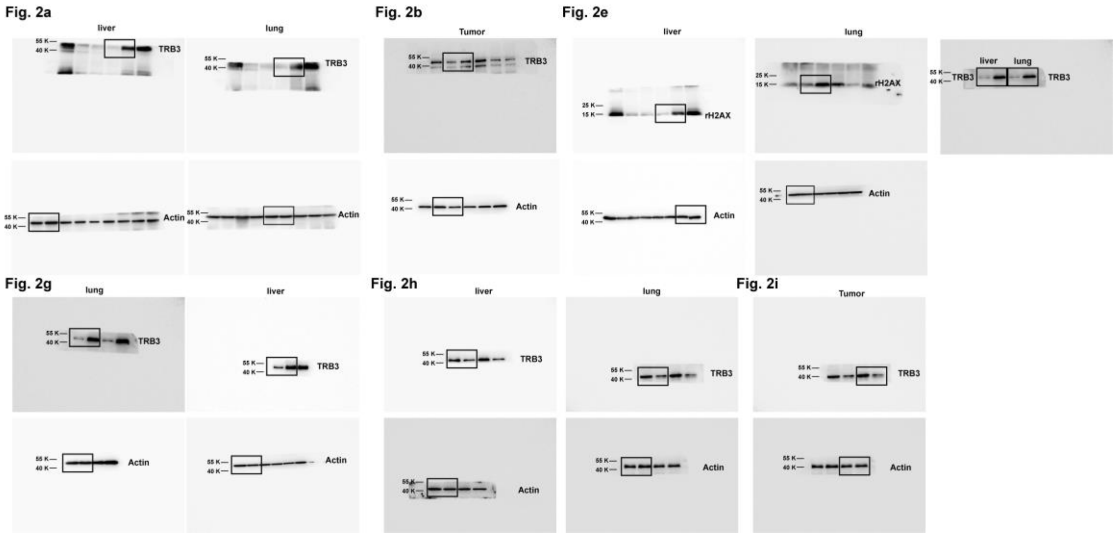


**Supplementary Figure 8. Uncropped blots used in Figure 2.**

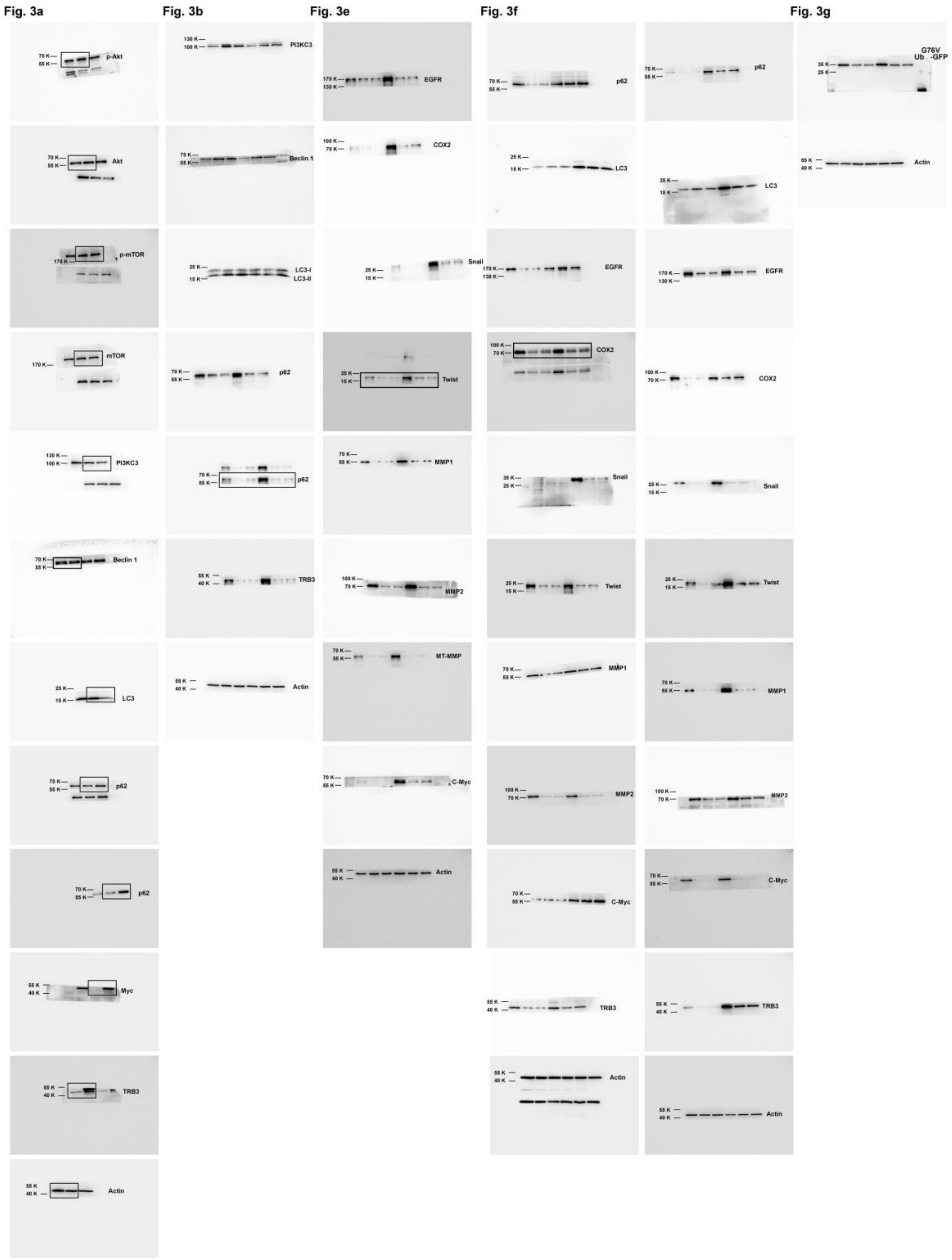


**Supplementary Figure 9. Uncropped blots used in Figure 3.**

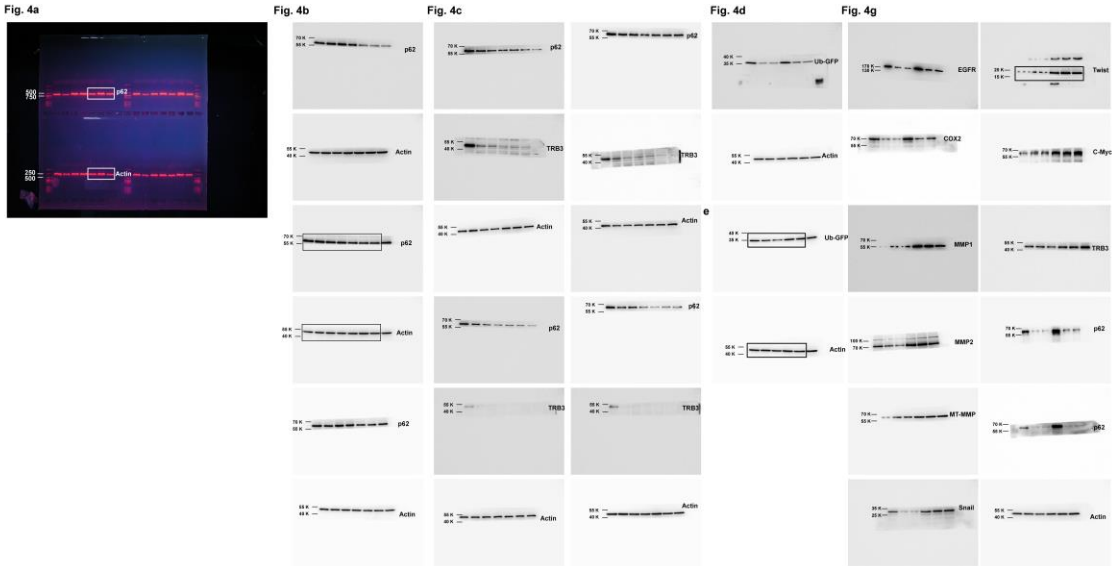


**Supplementary Figure 10. Uncropped blots used in Figure 4.**

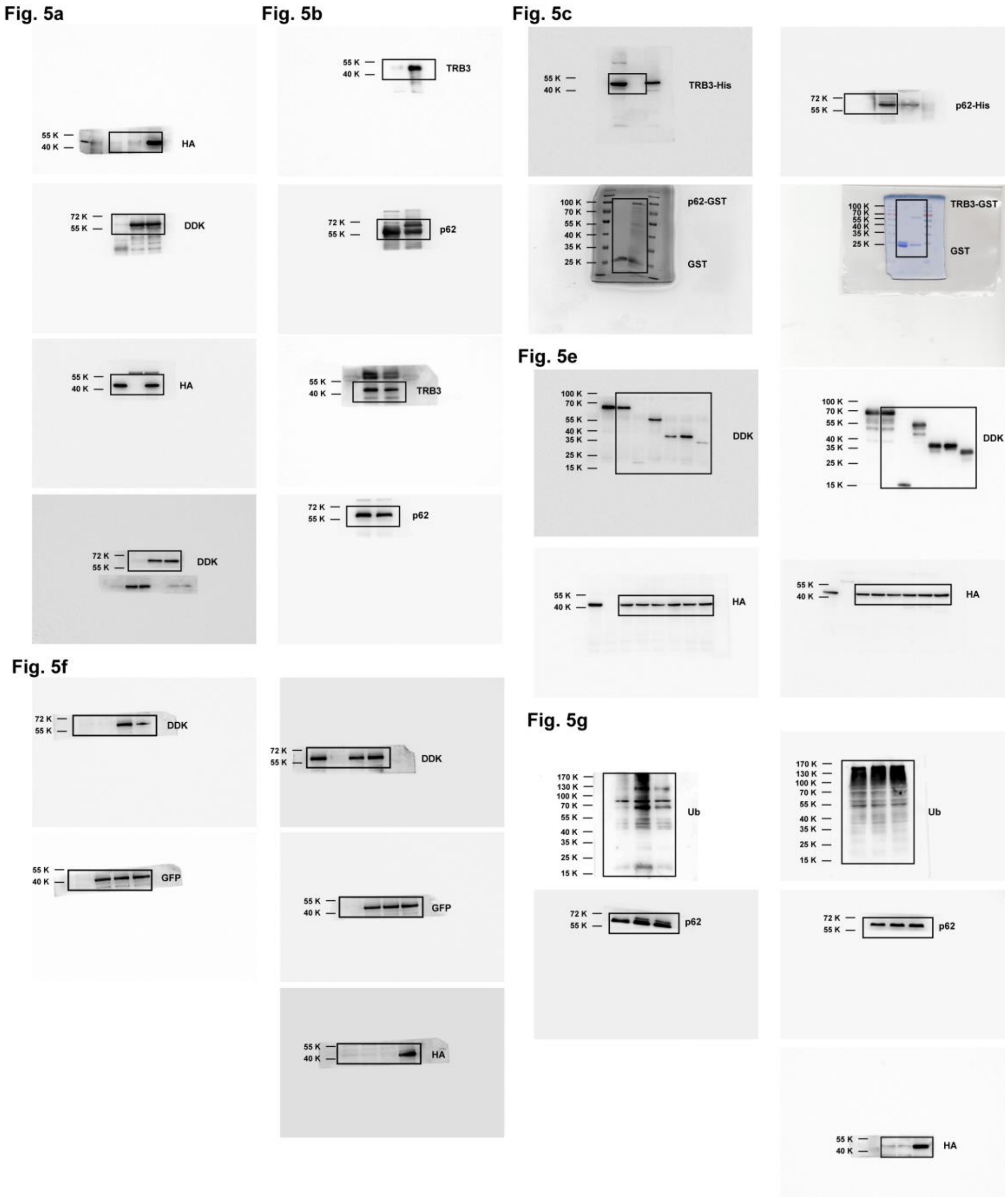


**Supplementary Figure 11. Uncropped blots used in Figure 5.**

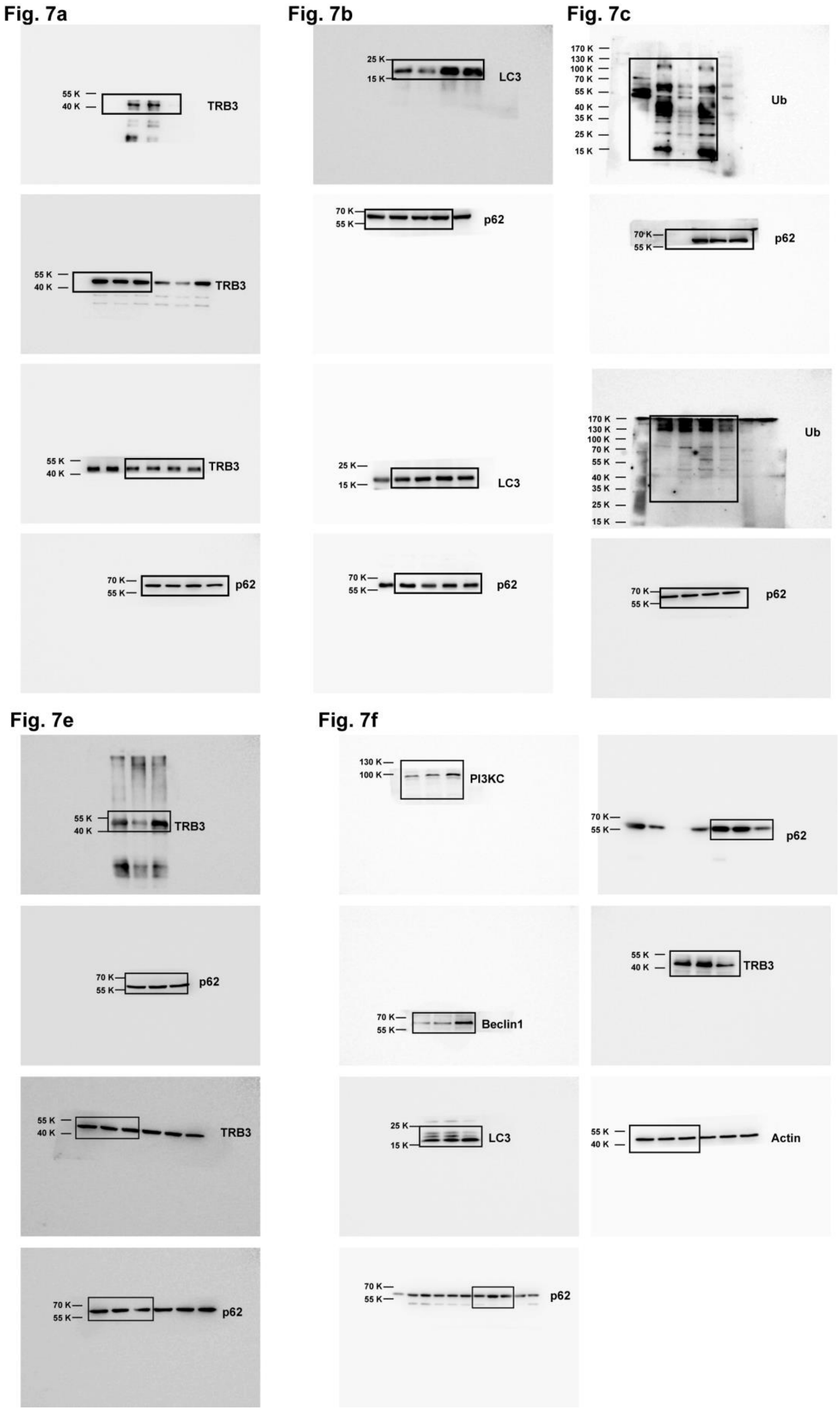


**Supplementary Figure 12. Uncropped blots used in Figure 7a-f.**

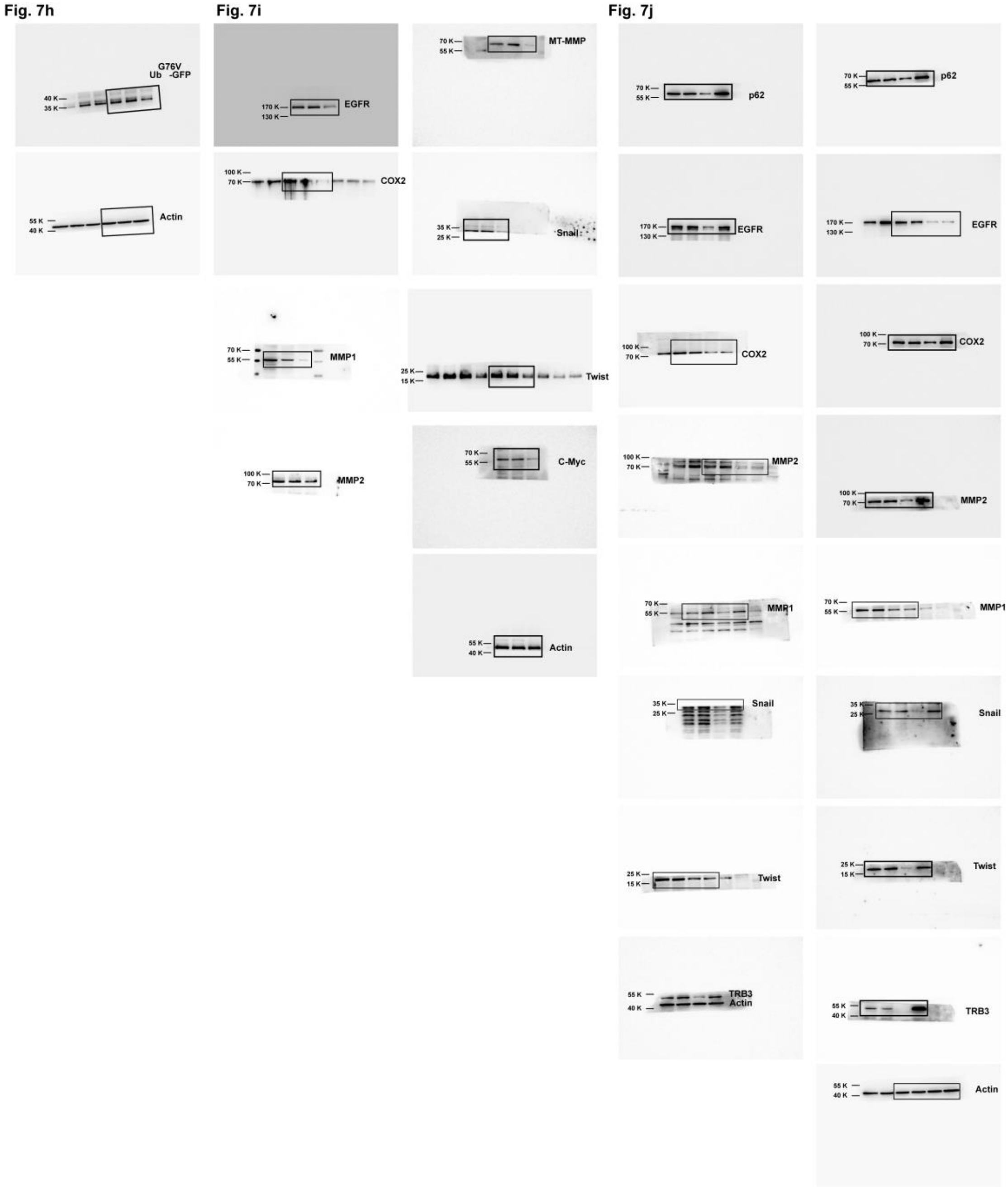


**Supplementary Figure 13. Uncropped blots used in Figure 7h-j.**

Fig. 8e

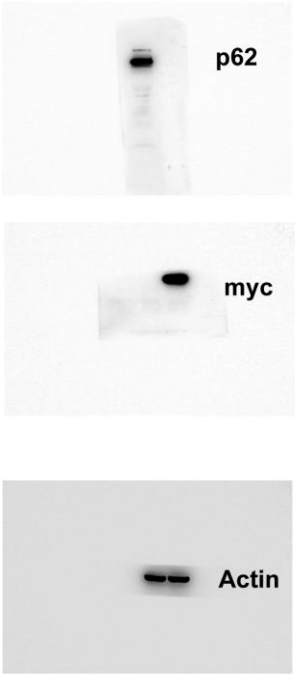


**Supplementary Figure 14. Uncropped blots used in Figure 8.**

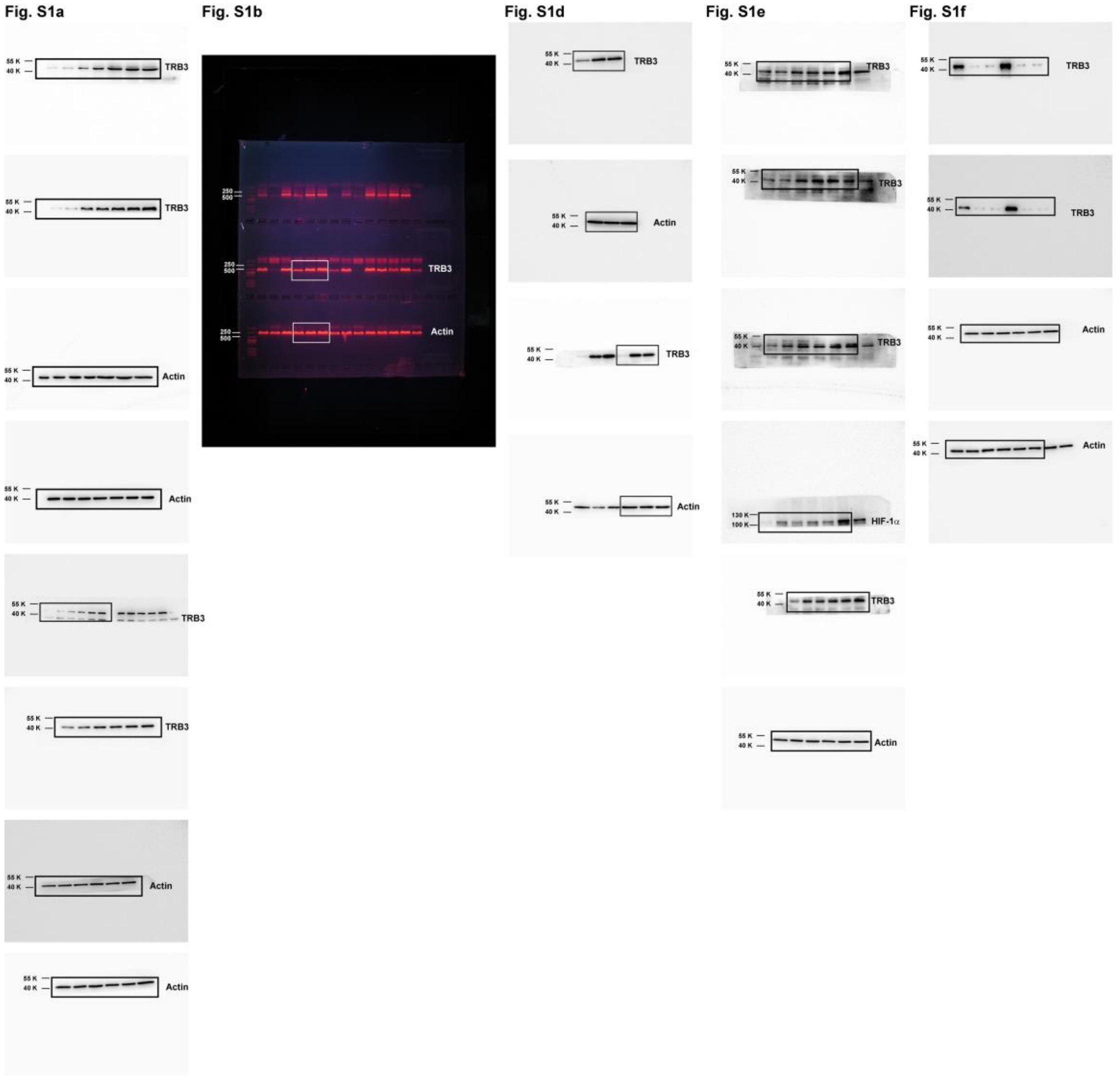


**Supplementary Figure 15. Uncropped blots used in Supplementary Figure 1a-f.**

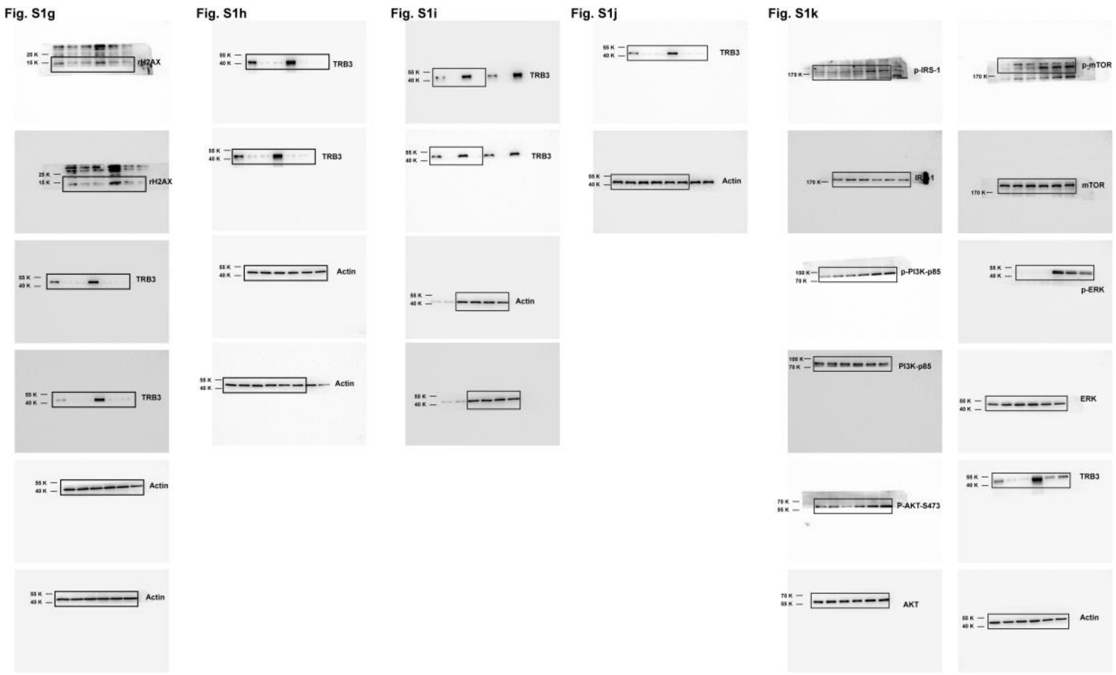


**Supplementary Figure 16. Uncropped blots used in Supplementary Figure 1g-k.**

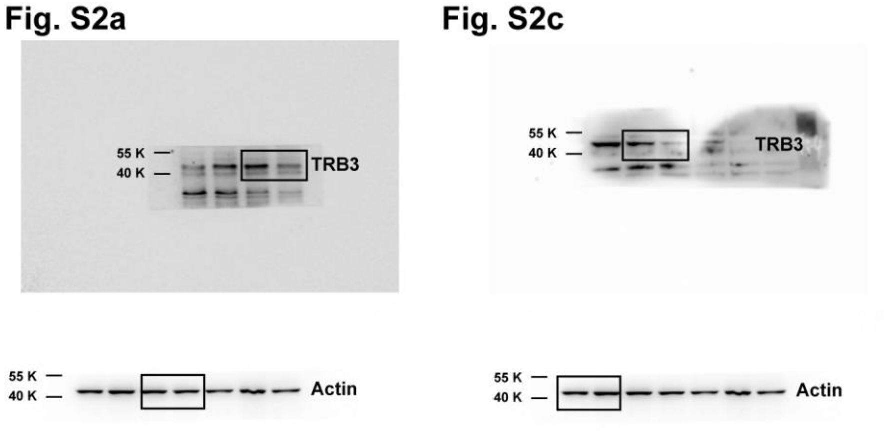


**Supplementary Figure 17. Uncropped blots used in Supplementary Figure 2.**

Fig. S4b

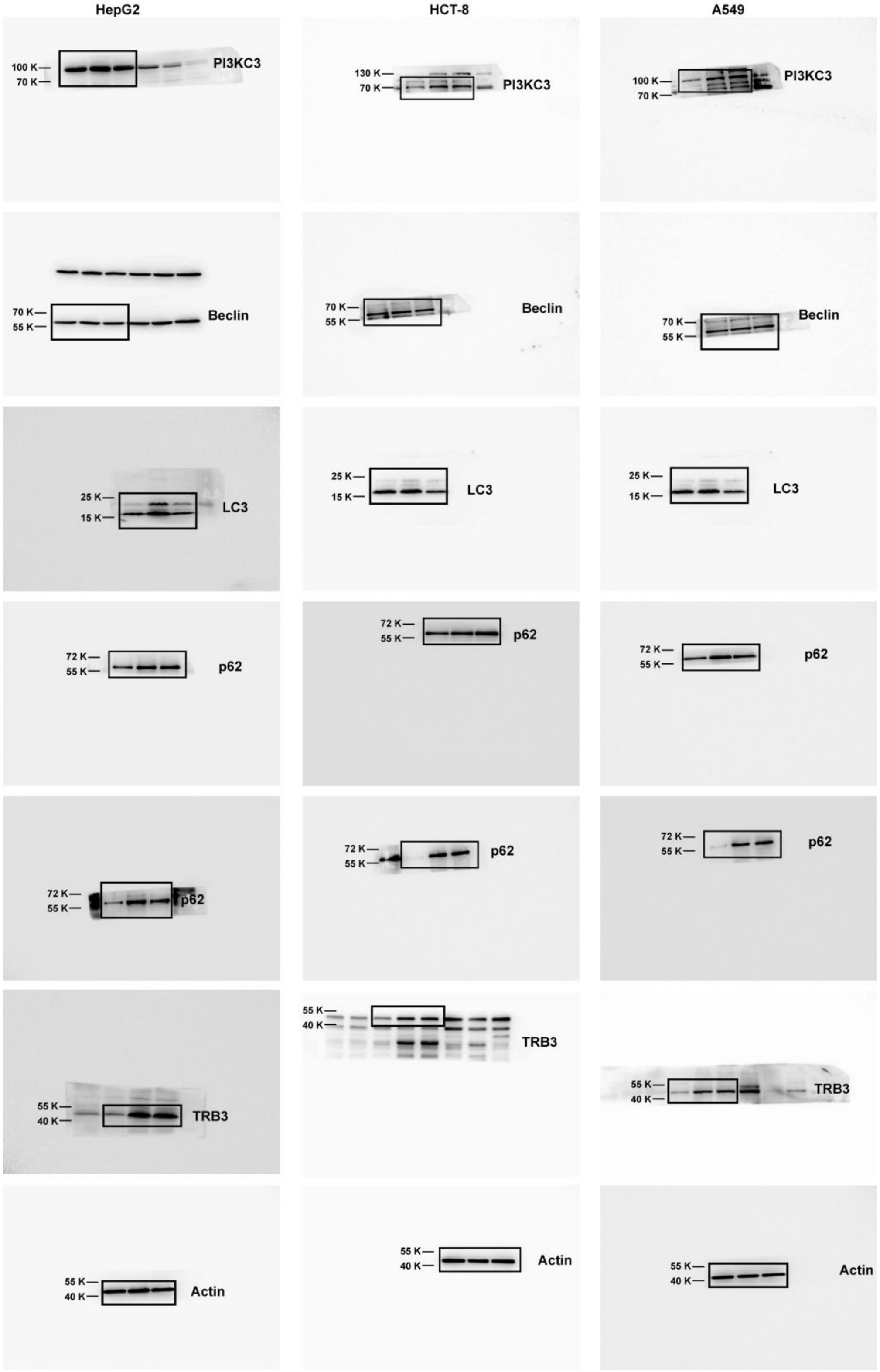


**Supplementary Figure 18. Uncropped blots used in Supplementary Figure 4b.**

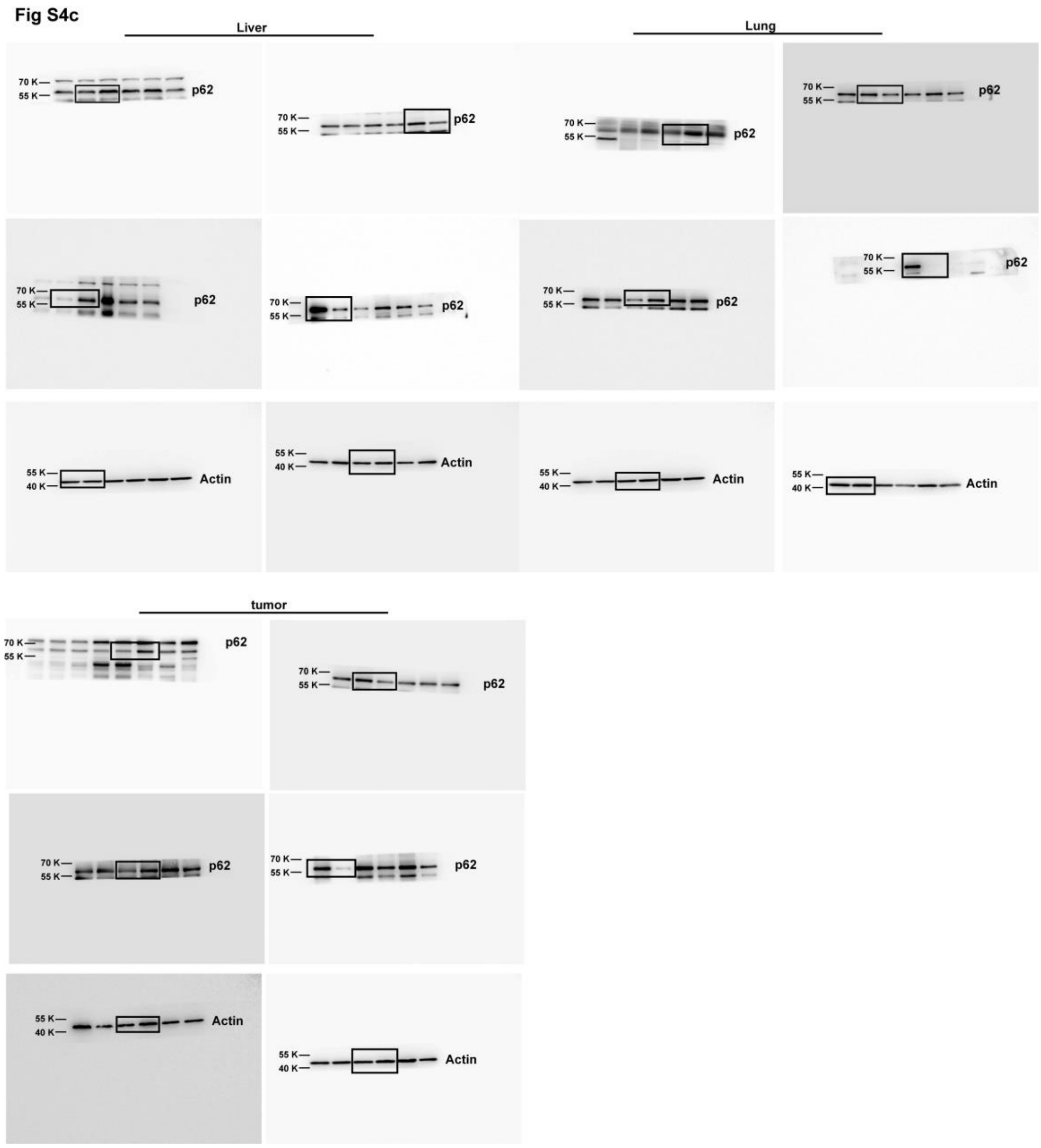


**Supplementary Figure 19. Uncropped blots used in Supplementary Figure 4c.**

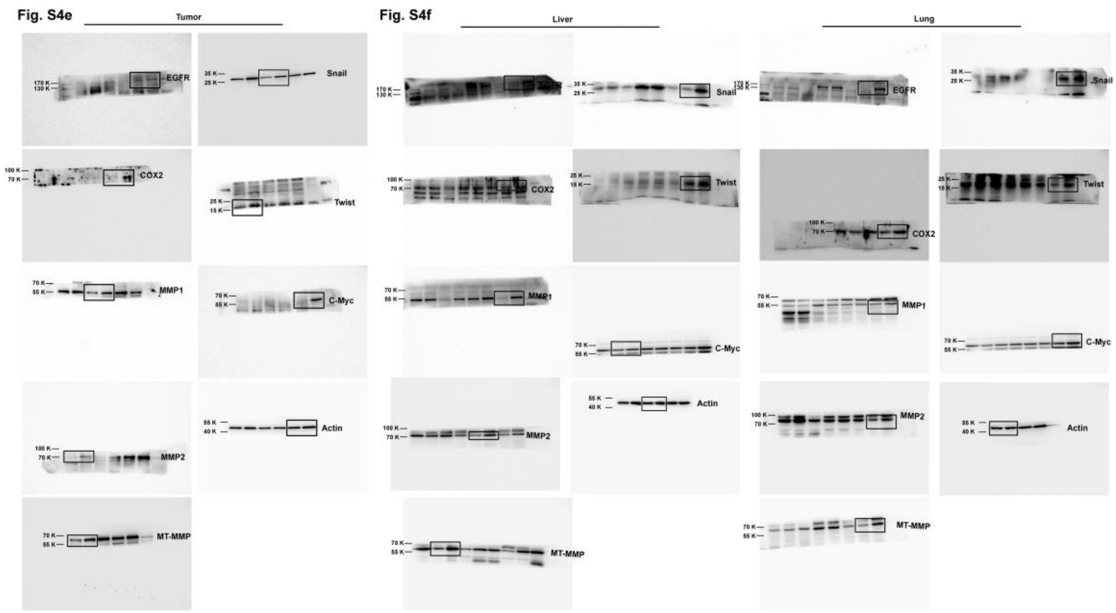


**Supplementary Figure 20. Uncropped blots used in Supplementary Figure 4e-f.**

Fig. S5a

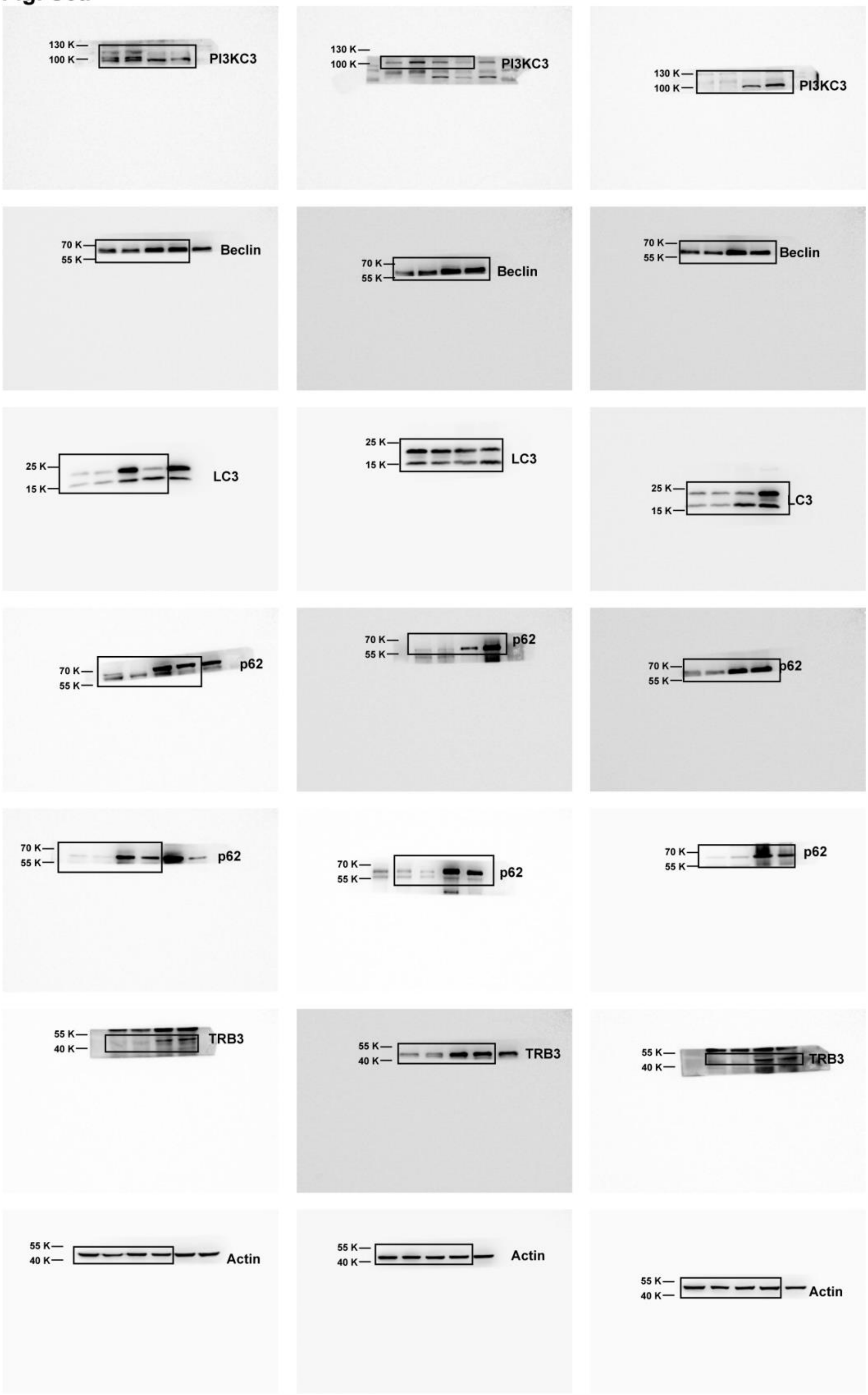


**Supplementary Figure 21. Uncropped blots used in Supplementary Figure 5a.**

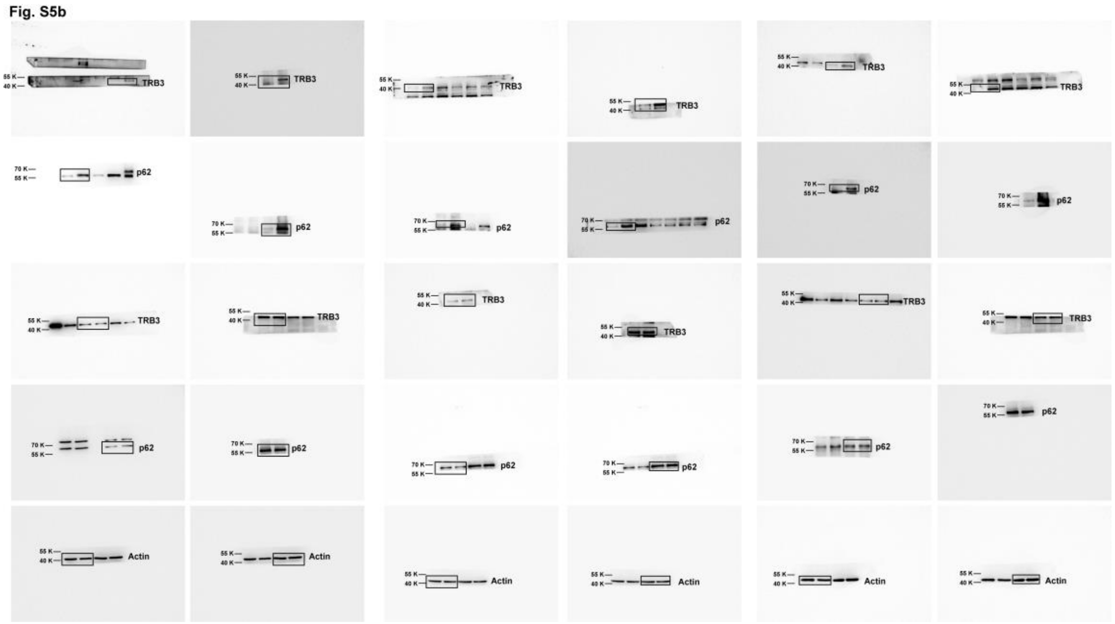


**Supplementary Figure 22. Uncropped blots used in Supplementary Figure 5b.**

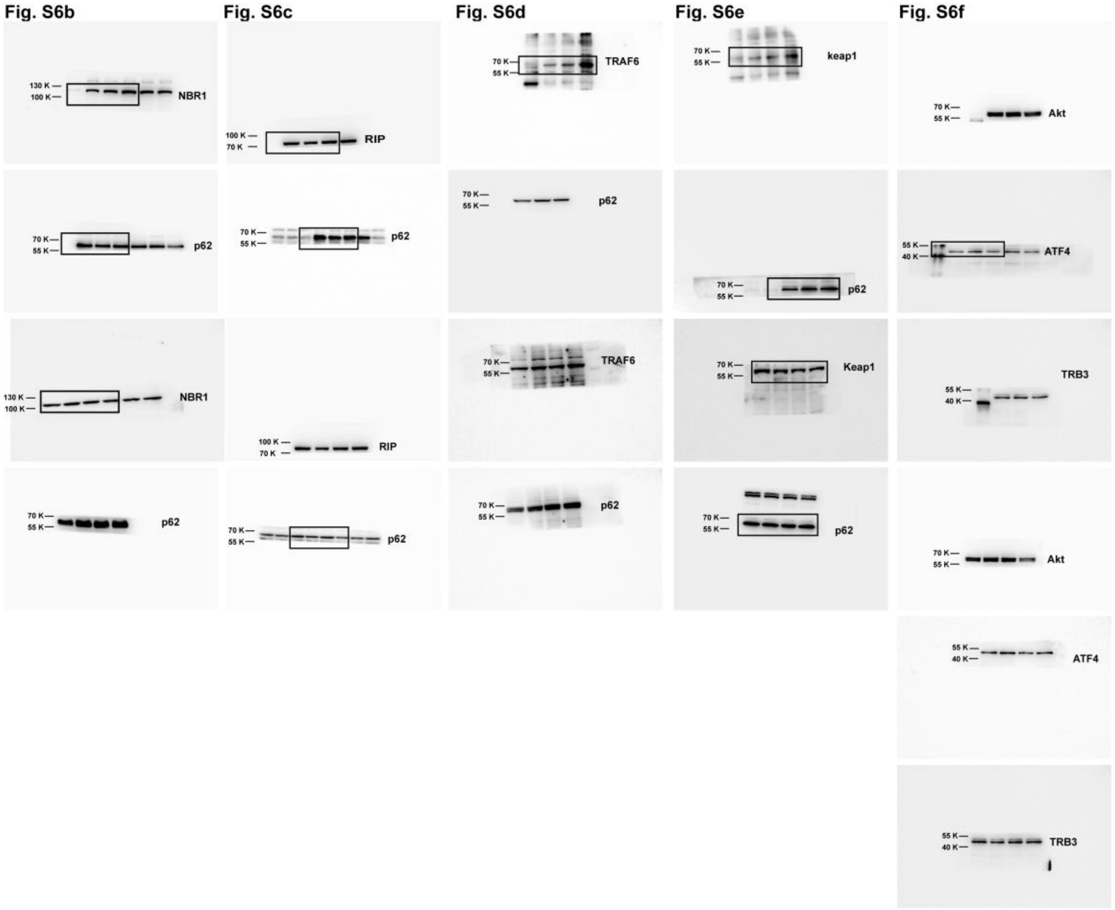


**Supplementary Figure 23. Uncropped blots used in Supplementary Figure 6.**

**Supplementary Table 1. Correlation between TRB3 and pIRS protein expression levels in HCC, colon cancer and lung cancer.**

| | HCC *n*=71 | | Colon cancer *n*=69 | | Lung cancer *n*=65 | |
|---|---|---|---|---|---|---|
| Protein expression | TRB3 | pIRS | TRB3 | pIRS | TRB3 | pIRS |
| Pearson r | 0.2721 | | 0.283 | | 0.34 | |
| *P* value (two-tailed) | 0.0217 | | 0.023 | | 0.004 | |
| *P* value summary | * | | * | | ** | |

**Supplementary Table 2. Multiorgan metastasis of B16-F10 melanoma cells injected intravenously in C57 BL/6 and diabetic KK-Ay mice.**

| Group | Tissue classes | | | | | |
|---|---|---|---|---|---|---|
| | Mesentery | Omentum | Lung | Axillary Lymph Node | Mediastinum | Kidney |
| C57 BL/6 | 0.00% | 0.00% | 50.00% | 0.00% | 8.33% | 0.00% |
| KK-Ay | 45.00% | 18.00% | 63.00% | 36.00% | 27.00% | 9.09% |
| KK-Ay/control | 0.00% | 1.25% | 62.50% | 25.00% | 50.00% | 12.50% |
| KK-Ay/TRB3-KD | 0.00% | 0.00% | 57.10% | 0.00% | 42.80% | 0.00% |

*****Each Value shows the percentage of multiorgan metastasis.

**Supplementary Table 3. Global protein changes observed in HepG2 cells expressing control-shRNA versus HepG2 cells expressing TRB3-shRNA1.**

Supplementary Table 3a

| Fold change>1.2<br>Control-shRNA/TRB3-shRNA1 | | |
|---|---|---|
| Cancer-promoting factor | Cancer-inhibiting factor | Others |
| EGFR/ErbB1 | HADHA | CD30 Ligand/TNFSF8 |
| OSM | IL-9 | CD30/TNFRSF8 |
| Glypican 5 | CD40 Ligand / TNFSF5 /CD154 | IL-4 |
| Frizzled-7 | Dkk-3 | Siglec-5/CD170 |
| IL-5 | Granzyme A | HCR / CRAM-A/B |
| IL-11 | FADD | IL-2 R beta /CD122 |
| FGF-18 | HSP60 | RELM alpha |
| Thrombopoietin (TPO) | | IL-2 R gamma |
| MMP-3 | | CD14 |
| CXCR4 (fusin) | | DR6 / TNFRSF21 |
| GM-CSF | | IFN-gamma |
| BMP-5 | | HCC-4 / CCL16 |
| Frizzled-3 | | Prolactin |
| EDG-1 | | IL-2 |
| Endothelin | | IL-3 |
| 6Ckine | | NT-3 |
| S100A6 | | FGF-13 1B |
| VEGF-D | | IL-10 R alpha |
| IL-17 | | ICAM-5 |
| Insulin | | IL-18 R alpha /IL-1 R5 |
| GRO-a | | |
| CCL28 / VIC | | |
| p21 | | |
| GRO | | |
| GDF5 | | |
| FGF-17 | | |
| FGF-5 | | |
| PTHLP | | |
| GDF1 | | |
| HSP90 | | |
| Frizzled-4 | | |
| MCP-1 | | |
| LRG1 | | |
| Frizzled-1 | | |

| | | |
|---|---|---|
| GCSF | | |
| GDF9 | | |
| Cathepsin B | | |
| IL-1 sRI | | |
| E-Selectin | | |
| VEGF R3 | | |
| Angiopoietin-like Factor | | |

Supplementary Table 3b

| Fold change < 0.8<br>Control shRNA / TRB3 shRNA1 | | |
|---|---|---|
| Cancer-promoting factor | Cancer-inhibiting factor | Others |
| GATA-4 | SIGIRR | CD36 |
| TRPM7 | AMPKa1 | OX40 Ligand / TNFSF4 |
| MMP-20 | WIF-1 | ApoE3 |
| Neuritin | Cadherin-13 | Cytokeratin 8 |
| AFP | ADAMTS-1 | IL-1 F8 / FIL1 eta |
| APN | CRTAM | Calsyntenin-1 |
| ANGPTL3 | TNF-beta | 11b-HSD1 |
| CRP | Cytokeratin 18 | ADAMTS-17 |
| CEA | Afamin | ApoB100 |
| Glut3 | SSTR2 | Serpin A8 |
| CD44 | CNDP1 | GPBB |
| LPS | DEFA1/3 | Neurokinin-A |
| Ras | HOXA10 | Alpha 1 AG |
| FGF-11 | FOXN3 | 2B4 |
| FSH | CBP | SMDF / NRG1Isoform |
| GLP-1 | Calreticulin | BAF57 |
| S100 A8/A9 | APC | ESAM |
| MMP-15 | Alpha Lactalbumin | Apelin |
| ASPH | FABP3 | SDF-1 / CXCL12 |
| Factor XIII A | VWF | Thymopoietin |
| FGFR1 alpha | LECT2 | CTACK / CCL27 |
| Tie-1 | S100A8 | C-peptide |
| GRP | Presenilin 1 | GPR-39 |
| CD59 | Clusterin | Amylin |
| Aldolase A | FRK | VGF |
| CA 125 | KLF4 | FoxP3 |
| Apex1 | GADD45A | ADAMTS-15 |
| ADAM-9 | PTPRD | Procalcitonin |
| pro-MMP13 | IL-29 | C5/C5a |
| SHBG | GDF3 | ADAMTS-19 |
| CA 19-9 | Chromogranin A | GPI |

| | | |
|---|---|---|
| Mesothelin | E-Cadherin | ADAMTS-18 |
| hCGb | Defensin | cTnT |
| SPINK1 | Thrombospondin-1 | Fibrinopeptide A |
| ABL1 | EXTL2 | APCS |
| Aldolase C | Serpin A5 | CXCR2 / IL-8 RB |
| EphA6 | EphB3 | Growth Hormone (GH) |
| EphA4 | LTF | GMNN |
| Fyn | MTUS1 | Uromodulin |
| EphA3 | Caspase-8 | BNP |
| Thymidine Kinase-1 | IL-21 | Cytokeratin 19 |
| Kallikrein 10 | IL-21 R | C9 |
| CA 15-3 | BAI-1 | CCK |
| Desmin | VDUP-1 | ApoC1 |
| ALK | Vitamin D Receptor | MSHa |
| Layilin | Nesfatin | Ceruloplasmin |
| EphA7 | Cystatin A | LYRIC |
| IL-19 | TNK1 | ApoA4 |
| ENPP2 | Serpin A1 | NR3C3 |
| Kallikrein 2 | | INSRR |
| ACK1 | | CD71 |
| Gelsolin | | Endorphin Beta |
| KCC3 | | BCAM |
| NAIP | | C7 |
| CHI3L1 | | hCG alpha |
| Kallikrein 14 | | MATK |
| SCG3 | | IL-12 p70 |
| S-100b | | Corticosteroid-binding globulin |
| NF1 | | EV15L |
| Pancreastatin | | CK-MB |
| Calbindin | | TRA-1-81 |
| MINA | | TSH |
| Lyn | | CART |
| VE-Cadherin | | PSP |
| HE4 | | Thyroglobulin |
| XIAP | | TRAIL R1 / DR4 / TNFRSF10A |
| CA 9 | | Cystatin C |
| BMX | | GST |
| PI 3Kinase p85 beta | | LTK |
| FAK | | LH |
| EphB2 | | Itk |
| FER | | Troponin C |
| EphA5 | | C8B |
| TRA-1-60 | | FIH |
| VIP Receptor 2 | | gamma-Thrombin |

| | | |
|---|---|---|
| uPAR | | BD-1 |
| Inhibin A | | ProSAAS |
| EphA8 | | Creatinine |
| Endothelin Receptor A | | SERPING1 |
| Btk | | POMC |
| Protein p65 | | Angiopoietin-like 2 |
| Complement factor H | | Marapsin |
| CD46 | | NPTX1 |
| Calcitonin | | NELL2 |
| PSA-total | | FGFR1 |
| PAI-1 | | TXK |
| GRP75 | | TPA |
| Fen 1 | | Tec |
| Somatotropin | | pro-Glucagon |
| Hck | | Thrombin |
| Nanog | | GFR alpha-4 |
| NET1 | | SRMS |
| HSP10 | | PPARg2 |
| IGF-II R | | TRPC6 |
| Trappin-2 | | NRG3 |
| SART1 | | |
| IL-1 R8 | | |
| Prohibitin | | |
| Insulin R | | |
| Omentin | | |
| INSL3 | | |
| ZAP70 | | |
| PSA-Free | | |
| Tyk2 | | |
| cIAP-2 | | |
| TYRO10 | | |
| HSP27 | | |
| Ferritin | | |
| COCO | | |
| ROR2 | | |
| ACTH | | |
| Kallikrein 11 | | |
| Mammaglobin A | | |
| CD74 | | |
| MIF | | |
| Erythropoietin R | | |
| IL-13 | | |
| ROR1 | | |
| TRKB | | |
| RYK | | |

| | | |
|---|---|---|
| FGFR2 | | |

(a) Proteins with expression downregulated when silencing TRB3 (Control-shRNA/TRB3-shRNA1>1.2). (b)Proteins with expression upregulated when silencing TRB3 (Control-shRNA/TRB3-shRNA1<0.8).

**Supplementary Table 4. Summary of pathological information of human cancer specimens used for immunoblot analysis.**

| Sample NO. | Age | Gender | Tissue Type | Diagnosis | TNM |
|---|---|---|---|---|---|
| 1 | 27 | Female | liver | Normal liver tissue | |
| 2 | 30 | Female | liver | Normal liver tissue | |
| 3 | 35 | Male | liver | Normal liver tissue | |
| 4 | 45 | Male | liver | Hepatocellular carcinoma, moderately differentiated | $T_2N_0M_0$ |
| 5 | 51 | Male | liver | Hepatocellular carcinoma, poorly differentiated | $T_3N_0M_0$ |
| 6 | 62 | Male | liver | Hepatocellular carcinoma, poorly differentiated | |
| 7 | 59 | Male | liver | Hepatocellular carcinoma, moderately differentiated | |
| 8 | 66 | Female | liver | Hepatocellular carcinoma, poorly differentiated | $T_4N_xM_x$ |
| 9 | 24 | Male | Colon | Normal colon tissue | |
| 10 | 25 | Male | Colon | Normal colon tissue | |
| 11 | 40 | Female | Colon | Normal colon tissue | |
| 12 | 75 | Male | Colon | Adenocarcinoma, well or moderately differentiated | $T_3N_0M_x$ |
| 13 | 41 | Male | Colon | Mucinous adenocarcinoma, poorly differentiated | $T_3N_XM_1$ |
| 14 | 72 | Male | Colon | Adenocarcinoma, moderately differentiated | $T_3N_0M_x$ |
| 15 | 61 | Female | Colon | Adenocarcinoma, well or moderately differentiated | $T_1N_0M_0$ |
| 16 | 50 | Male | Colon | Adenocarcinoma, moderately differentiated | $T_3N_0M_x$ |
| 17 | 28 | Male | Lung | Normal lung tissue | |
| 18 | 28 | Male | Lung | Normal lung tissue | |
| 19 | 25 | Female | Lung | Normal lung tissue | |
| 20 | 60 | Female | Lung | Adenosquamous carcinoma | $T_3N_0M_0$ |
| 21 | 52 | Female | Lung | Adenosquamous carcinoma | $T_4N_1M_0$ |
| 22 | 70 | Male | Lung | Small cell carcinoma and squamous cell carcinoma | $T_4N_1M_X$ |
| 23 | 64 | Male | Lung | Adenosquamous carcinoma, moderately differentiated | $T_4N_1M_1$ |
| 24 | 52 | Female | Lung | Papillary mucinous adenocarcinoma | $T_4N_1M_X$ |

Tissues and pathological informations were obtained from Alenabio (Xian, China)

## Supplementary Table 5. Multiorgan metastasis of B16-F10 melanoma cells injected intravenously in C57 BL/6 and diabetic KK-Ay mice.

| Group | Tissue classes | | | | | |
|---|---|---|---|---|---|---|
| | Mesentery | Omentum | Lung | Axillary Lymph Node | Mediastinum | Kidney |
| C57 BL/6 treated by Pep2-con | 0.00% | 0.00% | 33.3% | 0.00% | 25.0% | 0.00% |
| C57 BL/6 treated by Pep2-A2 | 0.00% | 0.00% | 16.7% | 0.00% | 25.00% | 0.00% |
| KK-Ay treated by Pep2-con | 27.2% | 27.2% | 54.5% | 27.2% | 9.09% | 9.09% |
| KK-Ay treated by Pep2-A2 | 18.1% | 9.09% | 0.00% | 0.00% | 0.00% | 0.00% |

*****Each Value shows the percentage of multiorgan metastasis.

## Supplementary Table 6. All primary antibodies against the indicated proteins used in the present study are listed.

| Antibody | Provider | Host | Dilution |
|---|---|---|---|
| HA-tag | MBL (561) | Rabbit | 1:1000 WB |
| DDK-tag | MBL (PM020, M185-3) | Mouse/Rabbit | 1:1000 WB |
| Myc-tag | MBL (M047-3) | Mouse | 1:1000 WB |
| GFP-tag | MBL (M048-3) | Mouse | 1:1000 WB |
| LC3 | Sigma (L7543) | Mouse | 1:100 IF |
| TRB3 | OriGene (TA303408) | Rabbit | 1:1000 WB<br>1:100 IHC |
| TRB3 | Abcam (ab88332) | Mouse | 1:100 IF |
| p62 | Sigma (P0067) | Rabbit | 1:5000 WB |
| p62 | Abcam (ab56416) | Rabbit | 1:500 IF |
| actin | Cell Signaling Technology (12262) | Mouse | 1:1000 WB |
| beclin 1 | Cell Signaling Technology (3495) | Rabbit | 1:1000 WB |
| p-Akt | Cell Signaling Technology (9271) | Rabbit | 1:1000 WB |
| Akt | Cell Signaling Technology (4691) | Rabbit | 1:1000 WB |
| mTOR | Cell Signaling Technology (2972) | Rabbit | 1:1000 WB |
| p-mTOR | Cell Signaling Technology (2971) | Rabbit | 1:1000 WB |
| PI3K p85 | Santa Cruz (sc-1637) | Rabbit | 1:1000 WB |
| pPI3K p85 | Cell Signaling Technology (4228) | Rabbit | 1:1000 WB |
| ERK | Cell Signaling Technology (4695) | Rabbit | 1:1000 WB |
| pERK | Cell Signaling Technology (4370) | Rabbit | 1:1000 WB |
| γH2AX | Cell Signaling Technology | Rabbit | 1:1000 WB |

| | | | |
|---|---|---|---|
| | (2577) | | |
| HIF-1α | Cell Signaling Technology (3716) | Rabbit | 1:1000 WB |
| PI3KC3 | Cell Signaling Technology (4263) | Rabbit | 1:1000 WB |
| C-Myc | Cell Signaling Technology (sc-40) | Rabbit | 1:1000 WB |
| COX2 | Cell Signaling Technology (12282) | Rabbit | 1:1000 WB |
| EGFR | Cell Signaling Technology (4267) | Rabbit | 1:1000 WB |
| MMP1 | Abcam (ab38929) | Rabbit | 1:1000 WB |
| MMP2 | Abcam (ab110186) | Rabbit | 1:1000 WB |
| MT-MMP | Abcam (ab53712) | Rabbit | 1:1000 WB |
| Ub | Cell Signaling Technology (3936) | Mouse | 1:250 IF |
| pTyr-100 | Cell Signaling Technology (9411) | Mouse | 1:1000 WB |
| Snail | Cell Signaling Technology (3879) | Rabbit | 1:1000 WB |
| Twist | Abcam (ab49254) | Rabbit | 1:1000 WB |
| pIRS-1 | Santa Cruz (sc-17196) | goat | 1:500 WB<br>1:100 IHC |
| IRS-1 | Cell Signaling Technology (2382) | Rabbit | 1:1000 WB |
